# Peptide‑based therapeutics targeting the SLC39A14‑PIWIL2 fusion in hepatocellular carcinoma

**DOI:** 10.1186/s44342-025-00060-5

**Published:** 2025-12-20

**Authors:** Masaud Shah, Sung Ung Moon, Ji-Hye Choi, Min Jae Kim, Hyun Goo Woo

**Affiliations:** 1 Department of Physiology, Ajou University School of Medicine, Suwon, 16499 Republic of Korea; 2 Department of Biomedical Science, Graduate School, Ajou University, Suwon, 16499 Republic of Korea; 3 Department of Pathology, Yonsei University College of Medicine, Seoul, Republic of Korea; 4 Ajou Translational Omics Center (ATOC), Research Institute for Innovative Medicine, Ajou University Medical Center, Suwon, Republic of Korea

**Keywords:** Fusion gene, HCC, PIWIL2, SLC39A14, Therapeutics-peptides

## Abstract

Fusion genes are key oncogenic drivers in various cancers; however, their role in hepatocellular carcinoma (HCC) remains underexplored. Here, we analyzed RNA-seq data from 68 HCC patients and identified several fusion products where SLC39A14-PIWIL2 stood out a putative driver. Functional assays revealed that the promoter of SLC39A14 potentially drives the overexpression of a truncated PIWIL2 protein (tPIWIL2), which retains its oncogenic MID and PIWI domains, in liver tissues. Both the wild-type and tPIWIL2 were found to interact with oncogenic partners HDAC3 and NME2 through these domains, as demonstrated by structural modeling and molecular dynamics simulations. To disrupt these interactions, we designed novel decoy peptides that potentially competes with both HDAC3 and NME2, effectively inhibiting PIWIL2-driven tumor activity in Huh7, HepG2, SNU449, and SNU398 HCC cell lines. Among the tested candidates, NEP1 markedly suppressed PIWIL2-driven oncogenic activity, and its co-administration with 5-fluorouracil (5-FU) significantly reduced PIWIL2-induced chemoresistance, thereby enhancing therapeutic efficacy. Collectively, these findings establish SLC39A14-PIWIL2 as a novel oncogenic fusion in HCC and highlight fusion protein–targeted peptide therapeutics as a promising avenue for precision treatment in HCC.

**Graphical Abstract:**

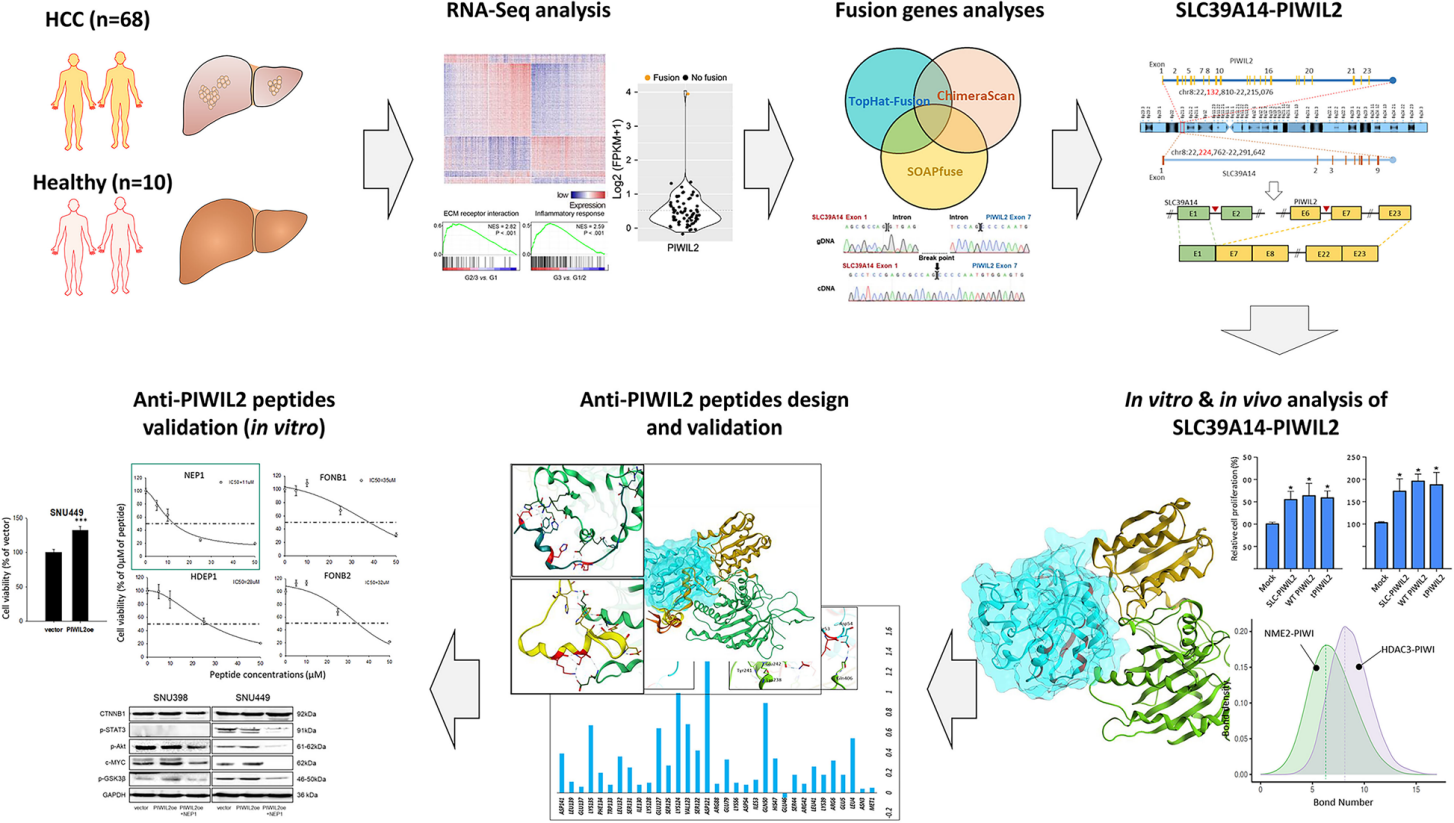

**Supplementary Information:**

The online version contains supplementary material available at 10.1186/s44342-025-00060-5.

## Introduction

Molecular and genetic complexity of hepatocellular carcinoma (HCC) impedes early diagnosis and limits treatment efficacy, thereby contributing to poor survival outcomes [1]. Advances in high-throughput genomic sequencing have unraveled the intricate molecular prerequisites of HCC, revealing its molecular heterogeneity with aberrations across various pathways, including inflammation, cell cycle control, invasion, metabolism, and metastasis [2–4]. In addition, chromosomal rearrangements and fusion genes stand out as critical oncogenic drivers across multiple cancer types [5–8]. These fusion genes serve as diagnostic biomarkers, such as ERG and ETV1, while others, such as ALK, RET, BRAF, and FGFR1–4, represent therapeutically targetable genes [9, 10]. In HCC, a limited number of fusion genes have been now identified with potential implications in tumor progression and prognosis. The DNAJB1-PRKACA fusion serves as a driver mutation in fibrolamellar carcinoma, a subtype of HCC [11]. The LINE1-MET fusion has also been highlighted in HCC development [12]. Furthermore, fusions such as SLC45A2-AMACR, ITCH-ASIP, and RNF138-RNF125 have been associated with better HCC prognoses, while MAN2A1-FER, CCNH-C5orf30, and SLC45A2-AMACR, detectable in serum, show potential for HCC diagnosis [13].

In this study, we identified a novel SLC39A14-PIWIL2 fusion gene in HCC patients’ samples. This fusion was formed by chromosomal inversion, joining SLC39A14 exon 1 with PIWIL2 exons 7–23, resulting in the upregulation of PIWIL2 and promoting HCC progression. Additionally, structural modeling of PIWIL2 and SLC39A14-PIWIL2, which encodes a truncated form (tPIWIL2), and their interactions with downstream proteins, alongside the design of PIWIL2 inhibitory peptides, has further highlighted its potential as a therapeutic target in HCC, opening avenues for the development of PIWIL2-focused treatments.

## Materials and methods

### Identification of fusion transcripts from RNA-seq data of HCC patient samples

The presence of fusion transcripts was identified from our previously published RNA-seq dataset (68 HCC and 10 non-tumor tissues; GSE113617) [14]. By applying three different methods of SOAPfuse [15], ChimeraScan [16], and TopHat-Fusion [17] with default parameters and filtering out transcripts with fewer than 30 reads at the breakpoints, we could identify 14 fusion transcripts which were detected by at least two of the methods.

### Human cancer cell lines, anticancer drug, and plasmids

The SNU398 (Seoul, Republic of Korea; Cat# 00398_SNU-398, RRID: CVCL_0077), SNU449 (KCLB Cat# 00449_SNU-449, RRID: CVCL_0454), HepG2 (KCLB Cat# 88065_HepG2, RRID: CVCL_0027), and Huh7 (KCLB Cat# 60104_Huh7, RRID: CVCL_0336) cell lines were purchased from Korean cell line bank and maintained in DMEM, MEM, or RPMI-1640 (Gibco BRL, Grand Island, NY) supplemented with 10% FBS and 1% antibiotics, at 37 °C in a 5% CO₂ incubator. PIWIL2, PIWIL2 without exon 1 (X1-PIWIL2) and SLC39A14-PIWIL2 constructs (tPIWIL2) were cloned into PCDNA3.1 (RRID: Addgene_70219) or C-terminal 3xFLAG-tagged PCDNA3.1 (RRID: Addgene_208616) using the In-Fusion cloning method (Clontech, Mountain View, CA). PCR products were amplified with CloneAmp HiFi PCR Premix (TAKARA, Tokyo, Japan) and specific primers (Table S1), followed by insertion into PCDNA3.1 plasmid using HindIII and ApaI. Constructs were confirmed by Sanger sequencing (Macrogen, Seoul, South Korea). The plasmids were transfected into the liver cancer cell lines (2 × 10^6^ cells per 60-mm dish) using 6 µg of Lipofectamine 3000 (Invitrogen, Thermo Fisher Scientific, Inc.), and incubated for 48 h at 37 °C in a CO₂ incubator. The cells expressing PIWIL2 were treated with various concentrations of 5-FU and peptides for 4 days in media supplemented with 10% FBS.

### Immunoblotting (western blotting)

Cells were harvested and lysed using lysis buffer (REF87787, Thermo Fisher Scientific), then centrifuged at ~ 13,000 × g for 10 min at 4 °C. Protein concentrations were determined with a Bradford protein assay kit (#5,000,006, Bio-Rad, Hercules, CA, USA). Equal amounts (30 µg) of protein were separated using 10% SDS-PAGE (Bio-Rad) and transferred to nitrocellulose membranes (#1,620,115, Bio-Rad) for immunoblotting. Membranes were washed three times with PBS (Welgene, Gyeongsangbuk-do, Republic of Korea) containing 0.1% Tween 20 (PBST; Sigma-Aldrich), blocked with PBST containing 1% bovine serum albumin (BSA, Bovogen, Melbourne, Australia) for 1 h at room temperature, and incubated with primary antibodies in PBST with 1% BSA overnight at 4 °C. After washing, membranes were incubated with secondary antibodies (1:1000 dilution) against goat anti-rabbit IgG-HRP (Cell Signaling Technology Cat# 7074S, RRID: AB_2099233) or anti-mouse IgG-HRP (Cell Signaling Technology Cat# 7076S, RRID: AB_330924) for 1 h at room temperature and washed again. Membranes were developed with ECL Buffer (REF34580, Thermo Fisher Scientific) and images captured using an iBright 1500 imaging system (REF34580, Thermo Fisher Scientific). The following antibodies were used: FLAG (1:1000, Sigma-Aldrich, Cat# F1804, RRID: AB_262044), CTNNB1 (1:1000, Santa Cruz Biotechnology, Cat# sc-7963, RRID: AB_626807), c-myc (1:1000, Cell Signaling Technology, Cat# 9402, RRID: AB_2151827), p-Akt (1:1000, Cell Signaling Technology Cat# 4060S, RRID:AB_2315049), p-GSK-3β (1:1000, Cell Signaling Technology, Cat# 9336S, RRID: AB_331405), p-STAT3 (1:1000, Cell Signaling Technology, Cat# 9145S, RRID: AB_2491009), β-actin (1:2000, Santa Cruz Biotechnology, Cat# sc-47778, RRID:AB_626632), and GAPDH (1:5000, Abcam Cat# ab8245, RRID: AB_2107448).

### Immunocytochemistry analysis

To examine c-myc localization, PIWIL2-overexpressing liver cancer cells were incubated with or without NEP1 peptide, fixed with 4% paraformaldehyde, and permeabilized with 0.25% Triton X-100. After washing, cells were blocked with 1% BSA and 0.1% Tween 20 for 1 h, then treated with primary antibodies against c-myc (1:100, Cell Signaling Technology, Cat# 9402, RRID: AB_2151827) and PIWIL2 (1:100, Abnova, Cat# MAB0843, RRID: AB_1204794) at 4 °C for 24 h. Following primary antibody incubation, cells were incubated with Alexa Fluor 594-conjugated donkey anti-rabbit IgG (1:200, Molecular Probes Cat# A-21207, RRID: AB_141637) and Alexa Fluor 488-conjugated donkey anti-mouse IgG (1:200, Molecular Probes Cat# A-21202, RRID: AB_141607) secondary antibodies for 2 h at room temperature. Nuclei were stained with DAPI-containing mounting solution, and cells were visualized under an Axiovert 200 fluorescence microscope (Carl Zeiss).

### RT-quantitative PCR analysis

Total RNA was isolated using RNeay (Qiagen, Hilden, Germany) for RT-qPCR analysis of target genes. RT-qPCR was performed using the iQ SYBR Green supermix (Bio-Rad, CA, USA) and the CFX96™ Real-Time system (Bio-Rad, Singapore). Reverse transcription was performed using TOPscript™ RT DryMIX (Enzynomics, Daejeon, Republic of Korea). The relative amounts of target genes were normalized to those of glyceraldehyde-3-phosphate dehydrogenase (GAPDH). The primer sets used are listed in table S1 in supplementary data. The 2^−ΔΔCq^ method was adopted to determine the fold changes (control vs. sample).

### Cell viability, spheroid formation, migration and invasion assay

For phenotypic changes, cells were seeded in 96-well plates (2 × 10^3^ cells/well) and incubated overnight at 37 °C with 5% CO₂. After transfection, 5 mg/mL MTT solution was added and incubated for 2 h. The blue precipitate was dissolved in 150 μl DMSO, and absorbance (550 nm) was measured using a microplate reader. All experiments were done in triplicate. For colony formation, cells were transfected for 48 h, then seeded at 500 cells/well in 6-well plates and incubated for 14 days. Colonies were washed, fixed with 3.7% paraformaldehyde, and stained with 1% crystal violet. Cell viability was assessed using the Cell Titer-Blue kit, and fluorescence intensity (555–585 nm, gain: 57) was measured using a SynergyHTX Fluorescent Microplate Fluorometer. For spheroids, cells were suspended in complete medium, seeded in ultra-low attachment plates, and incubated for 4 days. The spheroid viability was tested using the Cell Titer-Blue assay after trypsinization. Migration and invasion assays were performed in Transwells with 8-μm-pore filters, uncoated for migration or coated with matrigel for invasion. After incubation, non-migrated or non-invaded cells were removed, and the migrated or invaded cells were fixed, stained with crystal violet, and counted under microscope.

### Structural modeling, peptides design, and synthesis

While this study was ongoing, the cryo-EM structure of human PIWIL2 (Hili, PDB: 7YFX) was published [18]. However, we used the AlphaFold-predicted model in this study, as the two structures showed an overall root mean square deviation of 1.45 Å (Fig. S1A). Since both wild-type and tPIWIl2 retains MID and PIWI domains that bind HDAC3 and NME2, we used the wild-type model for docking analysis.

For NME2, we utilized its crystal structure (PDB: 7KPF) to analyze its binding with PIWIL2. This model contains the N-terminal hotspots that have been reported to bind PIWIL2 [19]. For HDAC3, the crystal structure (PDB: 4A69) lacks the C-terminal domain (aa 376–428) [20], which has been reported to exclusively bind the PIWI domain of PIWIL2 [21]. To add this motif into HDAC3, we retrieved the full-length HDAC3 model from the AlphaFold database for docking analysis [22, 23].

Protein–protein docking was performed using MOE, ClusPro and AlphaFold 3. AlphaFold generated high-confidence models using five random seeds and the interface were compared with that of previously reported hotspots [19, 21]. Models deviating from experimental results were excluded and the rest were ranked by AlphaFold’s predicted Local Distance Difference Test (pLDDT) and Predicted Aligned Error (PAE) scores. The final docking model was selected based on three criteria: (1) Lowest interface PAE (< 5 Å) and highest mean pLDDT (> 85); (2) Consistency of interface residues with previously reported binding hotspots [20, 31]; and (3) Thermodynamic stability confirmed by 100 ns molecular dynamics simulations (MDS). Models showing poor interface stability (fluctuation > 2 Å RMSF at the hotspot residues) or inconsistent interaction patterns were excluded. The final model used for analysis originated from the AlphaFold 3 were consistent in both interface accuracy and post-simulation stability. Final models underwent molecular dynamics simulations using GROMACS and binding free energy (BFE) calculations using MMPBSA methods [24]. We also employed MOE and ClusPro protein–protein docking platforms using AlphaFold-predicted peptide structures; however, the resulting docking poses were relatively inconsistent compared to those generated by AlphaFold 3.

Critical binding motifs within HDAC3 and NME2 contributing to BFE and interface stability were identified and subjected to in silico alanine mutagenesis [25]. These motifs served as templates to design synthetic decoy peptides (NEP1, NEP2, and HDEP1) targeting PIWIL2's interaction with HDAC3 or NME2, as described previously [25]. Peptide candidates were docked against PIWIL2 using alphaFold3, as described above.

All peptides were linked with CPP by their N-terminal and synthesized by GeneCust (Boynes, France) at a purity of over > 90%, as determined by reversed-phase high-performance liquid chromatography (HPLC; Shimadzu Prominence), described previously [25]. The HPLC reports and related information about peptide synthesis are provided in supplementary data (see Supplementary data).

### Statistics and reproducibility

All the statistical analysis was performed using SigmaPlot v12.5 software (Systat Software, Inc., San Jose, CA, USA), R packages (www.r-project.org), and GraphPad Prism (version 7). All experiments were performed in triplicate, and the data are represented as the mean ± standard deviation. *P < 0.05, **P < 0.01, and ***P < 0.001 compared to siNC or vector group. Significant difference was determined using the two-tailed Student’s t-test or One Way ANOVA Tukey test. *P* < 0.05 was considered statistically significant. Data were analyzed using SigmaPlot software (Systat Software, Inc.) to evaluate the two parameters (logistic three and quadratic) and determine the IC50 of the peptide or drug. The isobologram analysis [26] evaluates the nature of interaction of two drugs, i.e., drug A and drug B, at a given effect level.

Combination index (CI) is calculated as below:

$$\mathrm{CI}=\frac{{\mathrm C}_{\mathrm A,\mathrm x}}{{\mathrm{IC}}_{\mathrm x,\mathrm A}}+\frac{{\mathrm C}_{\mathrm B,\mathrm x}}{{\mathrm{IC}}_{\mathrm x,\mathrm B}}$$

A CI of less than, equal to, and more than 1 indicates synergy, additivity, and antagonism, respectively.

## Results

### Identification of SLC39A14-PIWIL2 fusion transcript in HCC

**Fig. 1:**
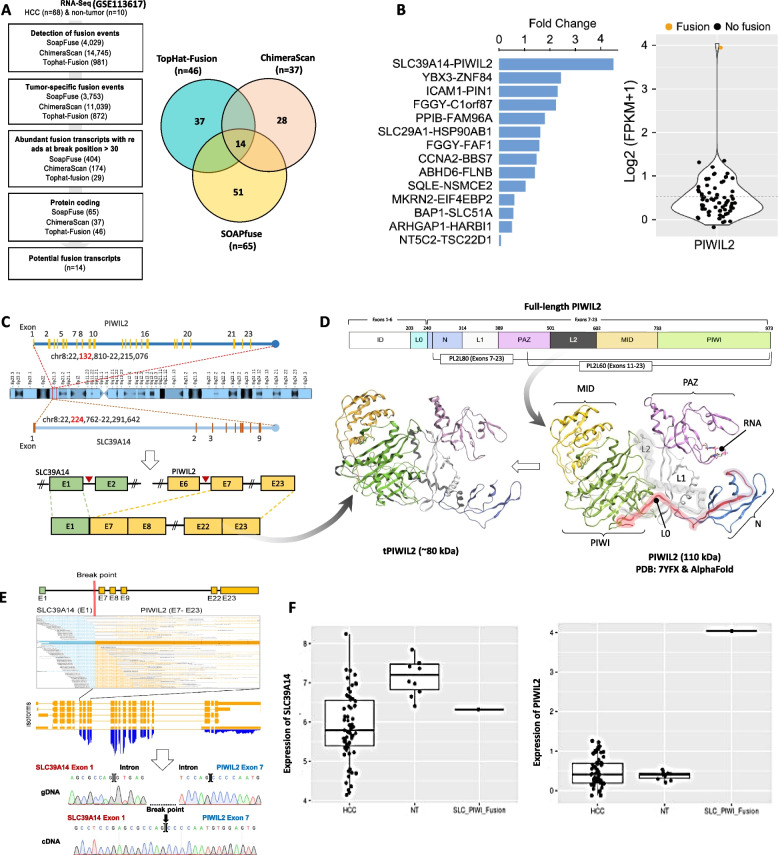
Identification of the SLC39A14-PIWIL2 fusion transcript in HCC. **A** Workflow of potential fusion transcripts identification using three different methods. **B** Bar plot showing the expression fold difference of the fusion products in HCC samples. PIWIL2 gene expression levels across 68 HCC patients. **C** Fusion structure of the SLC39A14-PIWIL2 transcript. **D** A 3D protein model of the full-length and tPIWIL2 proteins. **E** Aligned reads at the breakpoint of the SLC39A14-PIWIL2 fusion transcript in patient AJHCC007 (top). Sanger sequencing validations using genomic DNA (gDNA) and complementary DNA (cDNA) (bottom). **F** Boxplots showing the expression levels of SLC39A14 (left) and PIWIL2 (right) across three groups: HCCs without the SLC39A14-PIWIL2 fusion, HCCs with the fusion, and non-tumor samples

We used three methods—SOAPfuse, ChimeraScan, and TopHat-Fusion—to identify tumor-specific fusion transcripts with over 30 chimeric reads at the breakpoint involving protein-coding genes. Fourteen potential fusion transcripts were consistently detected by all methods (Fig. 1A). While no recurrent fusions were observed, we hypothesized that functional fusions might be expressed at higher levels than native transcripts. Among these, the SLC39A14-PIWIL2 fusion exhibited the highest expression (4.5-fold) relative to its native form (Fig. 1B). This fusion, caused by a chromosomal inversion on chromosome 8, joins exon 1 of SLC39A14 with exons 7–23 of PIWIL2 (Fig. 1C). Expasy Translate predicted a ~ 82.48 kDa PIWIL2 protein with exon 1 of SLC39A14 not contributing to the functional ORF (Fig. 1D, Fig. S2A). AlphaFold modeling revealed that the ~ 82.48 kDa PIWIL2 product lacks the intrinsically disordered (ID) region and the L0 motif in the N-terminal domain required for RNA binding but retains the PAZ, MID, and PIWI domains (Fig. 1D). We designated this product as truncated PIWIl2 “tPIWIL2”.

Sanger sequencing confirmed SLC39A14-PIWIL2 expression at the RNA level in the HCC sample (AJHCC007) but not at the genomic DNA level (Fig. 1E). Interestingly, SLC39A14 expression in HCC samples, including AJHCC007, was lower than in non-tumor samples, while PIWIL2 expression was markedly elevated in AJHCC007. However, SLC39A14 levels remained higher than PIWIL2 in other HCC and control samples, suggesting liver-specific induction (Fig. 1F, Fig. S2B) [27].

Using The Cancer Genome Atlas (TCGA), we identified SLC39A14-PIWIL2 fusions in stomach adenocarcinoma (STAD) and lung squamous cell carcinoma (LUSC), with breakpoints differing from those in AJHCC007 (Fig. S2C). In STAD, the fusion encodes full-length PIWIL2, while in LUSC, it produces a shorter PIWIL2 (530 aa, 60.7 kDa) similar to PL2L60, which promotes tumorigenesis via NF-κB [28]. Despite varying lengths, all forms are associated with tumorigenesis when expressed outside the testis.

### SLC39A14-PIWIL2 (tPIWIL2) promotes HCC progression

SLC39A14 maintains metal ion homeostasis in the liver and pancreas, resulting in higher expression in these organs compared to others, as indicated by the Human Protein Atlas dataset (Fig. S3A) [27]. Conversely, PIWIL2, a member of the PIWI subfamily of Argonaute proteins, is crucial for genome integrity during germ cell development and is predominantly expressed in the testis and duodenum, with minimal expression in liver tissues (Fig. S3B) [29]. These observations suggest that the rearrangement of SLC39A14 exon 1 into the 5' exon 7 of PIWIL2 likely enhances PIWIL2 expression in HCC. We hypothesized that tPIWIL2 expression in HCC is driven by the SLC39A14 promoter. RT-PCR analysis of the predicted promoter region (1000 bp) in AJHCC007 confirmed transcriptional activity, suggesting that the SLC39A14 promoter and exon 1 are rearranged into the 5′ region of PIWIL2 exon 7, inducing aberrant PIWIL2 expression (Fig. S3C).

**Fig. 2:**
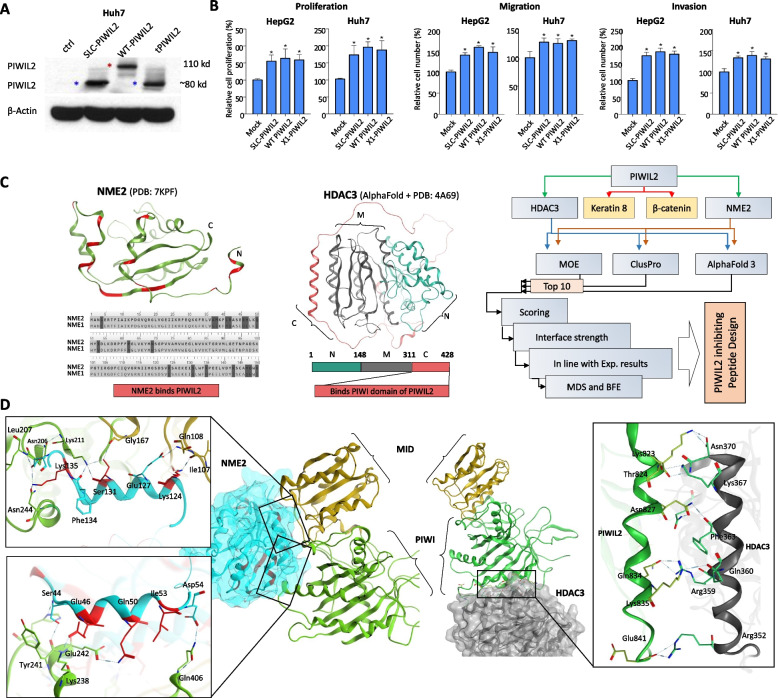
In vitro and in silico characterization of tPIWIL2 and protein–protein interaction. **A** Protein expression of the fused and non-fused forms of PIWIL2 proteins. **B** Phenotypic effects of PIWIL2 protein products on the Huh7 and HepG2 cell lines. **C** Interaction of PIWIL2 with NME2 and HDAC3. Residues in NME2 that differ from NME1 are highlighted in red within the aligned sequences. The PIWIL2-binding motif in HDAC3, located in the C-terminal region of the full-length protein, is also highlighted in red. An overview of the computational strategy used to predict interactions of PIWIL2 with its binding partners, HDAC3 and NME2, is shown. **D** The binding interface of MID-PIWI domains of PIWIL2 with NME2 (left) and PIWIL2 with HDAC3 (right) are shown

Since abnormal transcripts or proteins are often degraded via mechanisms like nonsense-mediated mRNA decay or the ubiquitin–proteasome system [30], we sought to determine whether SLC39A14-PIWIL2 produces a functional protein. We cloned and expressed WT PIWIL2, tPIWIL2, and X1-PIWIL2 (without exon 1) in Huh7 and HepG2 cells. RT-PCR and western blot analyses confirmed the expression of tPIWIL2 and X1-PIWIL2 at similar molecular weights (~ 80 kDa), consistent with exon 1 of SLC39A14 not contributing to the functional ORF (Fig. 2A, Fig. S3D). These results validate that SLC39A14-PIWIL2 encodes a functional PIWIL2 protein (tPIWIL2).

Aberrant PIWIL2 expression has been implicated in promoting proliferation in HCC and other cancers [19, 31], with its tumorigenic functions mediated through interactions with HDAC3, NME2, β-catenin (CTNNB1), and others via the PIWI and MID domains [19, 21, 28, 32]. In agreement, we demonstrated that tPIWIL2, like WT PIWIL2, enhances proliferation, invasion, and migration of liver cancer cells (HepG2 and Huh7, Fig. 2B). These findings confirm that the SLC39A14-PIWIL2 fusion transcript expresses an oncogenic tPIWIL2 protein, retaining its functional PIWI and MID domains.

### Structural insights into the PIWIL2 binding proteins, NME2 and HDAC3

To explore the downstream pathways contributing to the tumor-promoting functions of the PIWIL2 fusion product, we focused on two known PIWIL2-binding proteins, NME2 and HDAC3, both of which are implicated in oncogenic signaling—NME2 through its role in metastasis, proliferation, and nucleoside diphosphate kinase activity, and HDAC3 through epigenetic repression of tumor suppressor genes and promotion of cell survival. To gain structural insights into their interactions with PIWIL2, both molecules were docked with PIWIL2 (Fig. 2C). It is reported that NME2, but not its homolog NME1, binds PIWIL2 [19]. To understand this specificity, we aligned the sequences of NME1 and NME2, identifying critical amino acid differences that influence PIWIL2 binding (Fig. 2C, *left*). Structural analysis revealed that NME2 interacts with both the MID and PIWI domains of PIWIL2 via residues unique to NME2 (Fig. 2D, *left*). Meanwhile, HDAC3 binds exclusively to the PIWI domain of PIWIL2, utilizing a C-terminal helix motif consistent with previous excremental findings (Fig. 2D, *right*) [21].

**Fig. 3:**
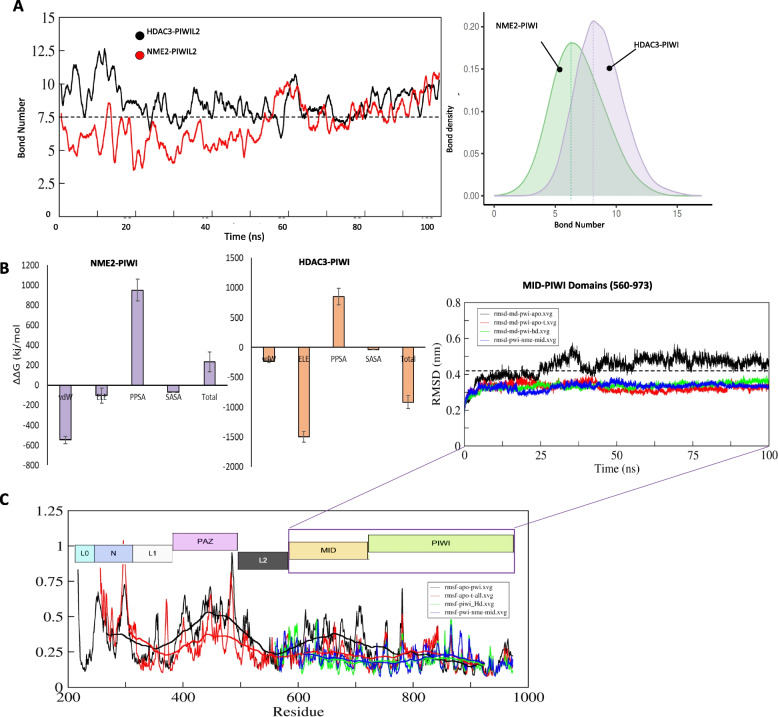
Structural modeling and stability evaluation of the PIWIL2 proteins. **A** Line and density plots showing changes in the number of hydrogen bonds of PIWIL2 with NME2 and HDAC3. **B** Binding free energy of PIWIL2 with NME2 and HDAC3 calculated through MMPBSA methods are depicted in a bar plots. **C** RMSF and RMSD plots of the PIWIL2 protein in complex with NME2 (nme) and HDAC3 (hd) or in its apo form (without binding partners) are shown

To evaluate the stability of these interactions, MDS were performed on the PIWIL2-NME2 and PIWIL2-HDAC3 complexes. BFE calculations and hydrogen bond density analyses demonstrated that both complexes maintained approximately 7.5 hydrogen bonds on average (Fig. 3A). Interestingly, BFE analyses revealed that PIWIL2 binds HDAC3 with greater affinity than NME2 (Fig. 3B), likely due to the higher number of electrostatic bonds observed in the PIWIL2-HDAC3 complex (Fig. 2D, *right*). Root mean square fluctuation analysis indicated that the N and PAZ domains of PIWIL2 exhibit higher flexibility compared to the MID and PIWI domains, with the latter being further stabilized upon binding to NME2 and HDAC3 (Fig. 3C). These findings underscore the importance of HDAC3 and NME2 in mediating PIWIL2's oncogenic functions, with interactions occurring primarily through the MID and PIWI domains, independent of the N-terminal and PAZ domains.

### PIWIL2 inhibiting peptides design and in vitro validation

**Fig. 4:**
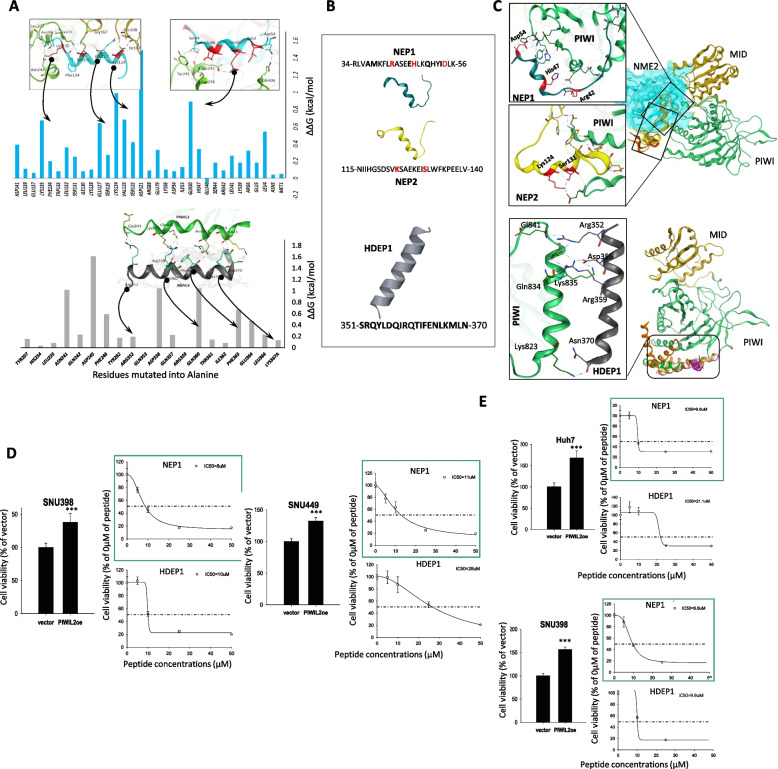
Anti-PIWIL2 peptides design and in vitro evaluation. **A** Hotspot residues identification in NME2 using in silico alanine scanning at the PIWIL2-NME2 interface (top) and PIWIL2-HDAC3 interface (bottom) are shown. **B** NME2-based PIWIL2-inhibiting peptides, NEP1 and NEP2 (top) and HDAC3-based PIWIL2-inhibiting peptide, HDEP1 are shown. **C** NEP1 and NEP2 peptides docked onto PIWIL2 (top) and HDEP1 peptide docked onto PIWIL2 (bottom) are shown. **D** Cell viability, is presented for PIWIL2-overexpressing liver cancer cell lines following treatment with peptides (NEP1, and HDEP1,). **E** Cell viability during sphere formation assays is shown for PWILL2-overexpressing liver cancer cell lines after treatment peptides

Given the critical role of the PIWIL2-HDAC3 and PIWIL2-NME2 binding in initiating oncogenic pathways, we used in silico alanine mutagenesis and identified two critical motifs in NME2 (amino acids 34–56 and 115–140) and a helical motif in HDAC3 (amino acids 351–370) that significantly contribute to the binding energies of their respective complexes (Fig. 4A). Based on these insights, we designed decoy peptides that could selectively disrupt these interactions, using decoy-based peptide design strategy (Fig. 4B). We have previously utilized this strategy in designing TLRs and SARS-CoV-2 inhibiting peptides [25, 33]. We found that all three peptides, NEP1, NEP2, and HDEP1, compete HDAC3 and NME2 for PIWIL2 binding, as suggested by the peptides clustering around MID and PIWI domains of PIWIL2 (Fig. 4C). This competitive binding is expected to attenuate the PIWIL2-mediated stabilization of HDAC3, the c-Myc regulatory function of NME2 and downstream oncogenic effects, such as cell proliferation, and modulation of critical signaling pathways like Wnt and Src/STAT3.

Using liver cancer cell lines (Huh7, HepG2, SNU449, SNU398) overexpressing PIWIL2 (Fig. S4A), we assessed peptide efficacy through cell viability and sphere formation assays. NEP1 consistently demonstrated the highest potency, achieving IC50 values of 8 μM in SNU398 and 11 μM in SNU449 for cell viability assays, and 9.6 μM (Huh7) and 8.6 μM (SNU398) in sphere formation assays. HDEP1 showed moderate activity, with IC50 values of 10 μM (SNU398) and 28 μM (SNU449) for cell viability, and 10 μM (Huh7) and 9.9 μM (SNU398) for sphere formation (Fig. 4D, E).

### NEP1 suppresses liver cancer oncogenesis by targeting the PIWIL2 pathway

**Fig. 5:**
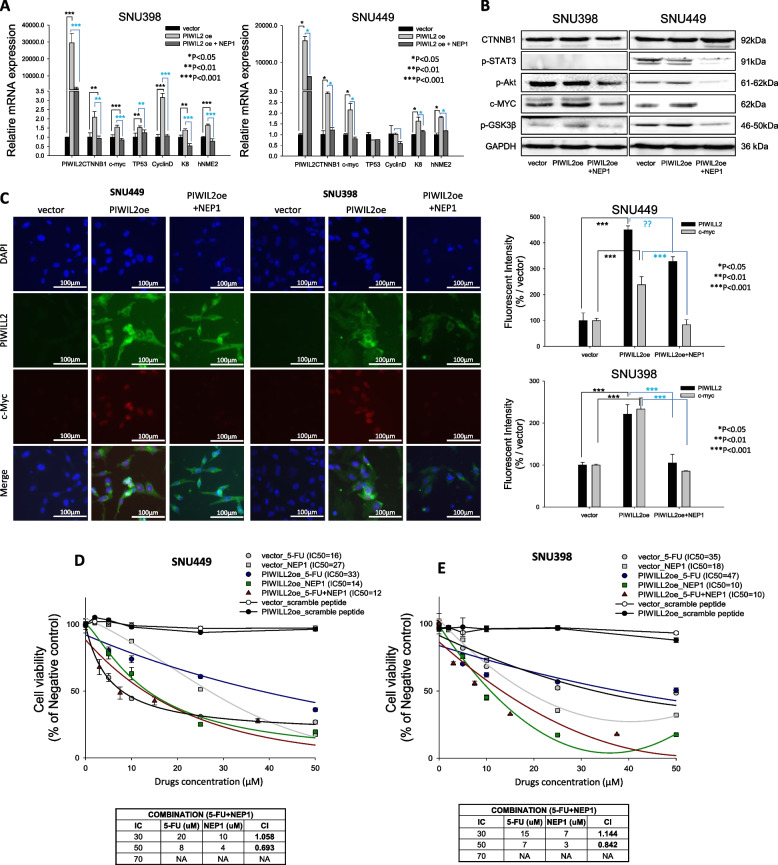
Therapeutic effect of PWILL2-inhibiting peptides. **A** RT-qPCR analysis showing the expression of PIWIL2, CTNNB1, c-myc, TP53, Cyclin D, K8, and hNME2 following treatment with NEP1 peptide. The effect of peptide was compared to that of vector-expressing and PIWIL-2-expressing peptide-untreated cells. Relative mRNA levels of target genes were normalized to GAPDH. **B** Immunoblotting of CTNNB1, p-STAT3, c-Akt, c-myc, and p-GSK3 under the same conditions. **C** Immunocytochemical analysis of c-myc localization was performed in PIWIL2-overexpressing cells. Fluorescence intensities were quantified and expressed as percentages relative to vector. Statistical significance is denoted as *P < 0.05, **P < 0.01, and ***P < 0.001 vs. vector. **D, E** To evaluate the synergistic effect of 5-FU and NEP1, cell viability assays were conducted and the Combination Index (CI) was determined in SNU449 (D) and SNU398 (E) cell lines. The control groups included cells treated with the vector alone followed by treatment with 5-FU, scramble peptide, or NEP1, as well as cells treated with both the PIWIL2-overexpressing plasmid and the scramble peptide. For CI analysis, IC30, IC50, and IC70 values were determined by treating cells with varying concentrations of 5-FU and NEP1 individually. These values were then compared to those obtained from simultaneous treatment with 5-FU and NEP1 at the varying concentrations, allowing for CI calculation. CI > 1 was defined as antagonism, CI = 1 as an additive effect, and CI < 1 as synergism

To investigate NEP1's effects on the PIWIL2 pathway, we analyzed RNA levels of PIWIL2-associated proteins in SNU398 and SNU449 cells. PIWIL2 overexpression significantly upregulated CTNNB1, c-myc, K8, and NME2 transcripts (Fig. 5A), while treatment with 10 μM NEP1 suppressed these changes, indicating NEP1's regulatory impact. Immunoblotting confirmed elevated p-AKT, GSK3β, and c-myc levels in PIWIL2-overexpressing cells, which NEP1 treatment restored to baseline (Fig. 5B). This aligns with prior findings that PIWIL2 knockdown reduces AKT and GSK3β phosphorylation [34]. Discrepancies in CTNNB1 mRNA and protein levels may result from p-GSK3β-mediated CTNNB1 stabilization, which increases protein accumulation without altering transcription [35–37]. Thus, RT-qPCR reflects CTNNB1 transcription, while immunoblotting captures post-translational regulation. Next, immunocytochemistry was performed to assess c-myc subcellular localization under PIWIL2 overexpression and NEP1 treatment. PIWIL2 caused significant nuclear accumulation of c-myc in both cell lines, which NEP1 effectively reversed (Fig. 5C). This aligns with previous findings that PIWIL2 interacts with NME2 to promote c-myc-driven proliferation in HeLa and HepG2 cells [19]. NEP1, in contrast, suppresses c-myc expression, inhibiting cell proliferation.

5-Fluorouracil (5-FU) is a pyrimidine analog that disrupts DNA and RNA synthesis [38] and is used to treat various cancers, including colorectal, breast, gastric, pancreatic, and head and neck malignancies [39]. We examined the combined effects of NEP1 and 5-FU on liver cancer cell line SNU449 (Fig. 5D) and SNU398 (Fig. 5E). PIWIL2 overexpression increased the IC50 of 5-FU but decreased the IC50 of NEP1. Co-administration of NEP1 and 5-FU at equal concentrations (0, 2, 5, 10, 25, and 50 µM) reduced the IC50 of the combination under PIWIL2 overexpression. CI analysis showed antagonism at IC30 but synergy at IC50, with CI values of 0.693 and 0.842 in SNU398 and SNU449, respectively. These findings suggest that NEP1 reverses PIWIL2-driven oncogenic changes, including c-myc nuclear accumulation and chemoresistance, and synergistically enhances 5-FU efficacy, supporting its potential as a targeted cancer therapy. This approach also highlights opportunities for personalized treatment strategies targeting specific molecular drivers.

## Discussion

Fusion-driven oncogenesis often involves promoter activation of oncogenes by tissue-specific or highly expressed genes. For example, TMPRSS2-ERG and SLC45A3-BRAF fusions in prostate cancer similarly amplify oncogene expression via highly active promoters [40]. The SLC39A14-PIWIL2 fusion identified in this study follows this mechanism, with the SLC39A14 promoter driving PIWIL2 overexpression, amplifying its tumor-promoting potential in HCC. The rearrangement activates the SLC39A14 promoter, which drives the expression of PIWIL2, an oncogene typically restricted to gonads [41], leading to low molecular weight PIWIL2 (tPIWIL2) overexpression. This promotes HCC progression by activating oncogenic pathways.

PIWIL2 promotes tumorigenesis through interactions with key proteins and pathways. It stabilizes HDAC3 via its PIWI domain by preventing degradation and enhancing phosphorylation by CK2α, promoting cell proliferation and suppressing apoptosis [21]. It interacts with NME2, supporting c-Myc-mediated oncogenesis [19], and binds CTNNB1 via its PAZ domain, implicating the Wnt signaling pathway [32]. Additionally, PIWIL2 inhibits apoptosis by forming a PIWIL2/K8/p38 complex, stabilizing K8, reducing Fas, and repressing p53 phosphorylation [42]. These multifaceted interactions make PIWIL2 a critical driver of oncogenesis and a promising therapeutic target.

The protein product of the SLC39A14-PIWIL2 fusion retains the oncogenic MID and PIWI domains of PIWIL2, driving proliferation, invasion, and migration in Huh7 and HepG2 cells despite losing the intrinsically disordered region and L0 motif. Interestingly, similar fusion transcripts were found in STAD and LUSC, with distinct breakpoints and functions. In LUSC, the truncated PIWIL2 isoform resembles PL2L60, associated with NF-κB activation and tumorigenesis [28], underscoring its potential as a universal therapeutic target. Our structural modeling revealed that tPIWIL2 interacts with HDAC3 and NME2 via its MID and PIWI domains, thereby potentially driving its aberrant oncogenicity in liver tissues. Therefore, key residues essential for these interactions were identified through molecular dynamics simulations and alanine mutagenesis, enabling the design of decoy peptides (NEP1 and HDEP1) to competitively disrupt these interactions.

Current HCC treatments, such as immune checkpoint inhibitors and multi-kinase inhibitors, face limitations due to tumor heterogeneity and resistance [43]. Targeting PIWIL2 through small molecules, RNA-based approaches, or peptide therapeutics offers a novel strategy for addressing these challenges. The peptides developed in this study effectively attenuate PIWIL2-mediated oncogenic signaling, demonstrating therapeutic potential. Moreover, the SLC39A14-PIWIL2 fusion could serve as a biomarker for early diagnosis and disease stratification. Detecting fusion transcripts in serum, as shown with other fusions like SLC45A2-AMACR [44], could enable minimally invasive diagnostic assays tailored to HCC.

### Limitations and future directions

We identified a rare case of SLC39A14-PIWIL2 expression in HCC, with evidence of its occurrence in other cancer types. Although the expression frequency of the fusion transcript is low, targeted therapies against this fusion may provide promising approach for managing patients harboring this alteration. In addition, this study establishes the oncogenic role of SLC39A14-PIWIL2 in HCC; however, further investigation is needed to elucidate its role in the tumor microenvironment and its interplay with immune evasion mechanisms. Future studies should also focus on optimizing the delivery and stability of PIWIL2-inhibiting peptides for in vivo applications. Although we partly evaluated (5-FU), combining these targeted therapies with existing treatment modalities, such as immune checkpoint inhibitors or kinase inhibitors, could provide synergistic benefits and overcome resistance mechanisms in advanced HCC.

## Supplementary Information

Supplementary Material 1. 

Supplementary Material 2. 

## Data Availability

The raw data of the genomic profiles are available in the GEO database (http://www.ncbi.nlm.nih.gov/projects/geo) under ac cession number GSE113617.

## References

[1] Suresh A, Dhanasekaran R. Implications of genetic heterogeneity in hepatocellular cancer. Adv Cancer Res. 2022;156:103–35. 35961697 10.1016/bs.acr.2022.01.007 PMC10321863

[2] Cancer Genome Atlas Research Network. Electronic address, w.b.e. and N. Cancer genome atlas research, comprehensive and integrative genomic characterization of hepatocellular carcinoma*.* Cell. 2017;169(7):1327–1341 e23. 10.1016/j.cell.2017.05.046 PMC5680778 28622513

[3] Cui D, et al. Advances in multi-omics applications in HBV-associated hepatocellular carcinoma. Front Med. 2021;8:754709. 10.3389/fmed.2021.754709 PMC8514776 34660653

[4] Huang W, AJ Skanderup, CG Lee. Advances in genomic hepatocellular carcinoma research*.* Gigascience. 2018;7(11):giy135. 10.1093/gigascience/giy135 PMC6335342 30521023

[5] Gainor JF, Shaw AT. Novel targets in non-small cell lung cancer: ROS1 and RET fusions. Oncologist. 2013;18(7):865–75. 23814043 10.1634/theoncologist.2013-0095 PMC3720641

[6] Rubin MA, Maher CA, Chinnaiyan AM. Common gene rearrangements in prostate cancer. J Clin Oncol. 2011;29(27):3659–68. 21859993 10.1200/JCO.2011.35.1916 PMC4874145

[7] Stenman G, et al. Chromosome translocations, gene fusions, and their molecular consequences in pleomorphic salivary gland adenomas. Biomedicines. 2022;10(8):1970. 10.3390/biomedicines10081970 PMC9405559 36009517

[8] Yu YP, et al. Identification of recurrent fusion genes across multiple cancer types. Sci Rep. 2019;9(1):1074. 30705370 10.1038/s41598-019-38550-6 PMC6355770

[9] Kumar-Sinha C, Kalyana-Sundaram S, Chinnaiyan AM. Landscape of gene fusions in epithelial cancers: seq and ye shall find. Genome Med. 2015;7:129. 26684754 10.1186/s13073-015-0252-1 PMC4683719

[10] Schram AM, et al. Fusions in solid tumours: diagnostic strategies, targeted therapy, and acquired resistance. Nat Rev Clin Oncol. 2017;14(12):735–48. 28857077 10.1038/nrclinonc.2017.127 PMC10452928

[11] Bauer J, et al. The oncogenic fusion protein DNAJB1-PRKACA can be specifically targeted by peptide-based immunotherapy in fibrolamellar hepatocellular carcinoma. Nat Commun. 2022;13(1):6401. 36302754 10.1038/s41467-022-33746-3 PMC9613889

[12] Kiyose H, et al. Comprehensive analysis of full-length transcripts reveals novel splicing abnormalities and oncogenic transcripts in liver cancer. PLoS Genet. 2022;18(8):e1010342. 35926060 10.1371/journal.pgen.1010342 PMC9380957

[13] Yu YP, et al. Detection of fusion transcripts in the serum samples of patients with hepatocellular carcinoma. Oncotarget. 2019;10(36):3352–60. 31164957 PMC6534357

[14] Yoon S, et al. USO1 isoforms differentially promote liver cancer progression by dysregulating the ER-Golgi network. Carcinogenesis. 2021;42(9):1208–20. 34293111 10.1093/carcin/bgab067

[15] Jia W, et al. SOAPfuse: an algorithm for identifying fusion transcripts from paired-end RNA-Seq data. Genome Biol. 2013;14(2):R12. 23409703 10.1186/gb-2013-14-2-r12 PMC4054009

[16] Iyer MK, Chinnaiyan AM, Maher CA. ChimeraScan: a tool for identifying chimeric transcription in sequencing data. Bioinformatics. 2011;27(20):2903–4. 21840877 10.1093/bioinformatics/btr467 PMC3187648

[17] Kim D, Salzberg SL. Tophat-Fusion: an algorithm for discovery of novel fusion transcripts. Genome Biol. 2011;12(8):R72. 21835007 10.1186/gb-2011-12-8-r72 PMC3245612

[18] Li Z, et al. Mammalian PIWI-piRNA-target complexes reveal features for broad and efficient target silencing. Nat Struct Mol Biol. 2024;31(8):1222–31. 38658622 10.1038/s41594-024-01287-6

[19] Yao Y, et al. PIWIL2 induces c-Myc expression by interacting with NME2 and regulates c-Myc-mediated tumor cell proliferation. Oncotarget. 2014;5(18):8466–77. 25193865 10.18632/oncotarget.2327 PMC4226697

[20] Watson PJ, et al. Structure of HDAC3 bound to co-repressor and inositol tetraphosphate. Nature. 2012;481(7381):335–40. 22230954 10.1038/nature10728 PMC3272448

[21] Zhang Y, et al. PIWIL2 suppresses Siah2-mediated degradation of HDAC3 and facilitates CK2alpha-mediated HDAC3 phosphorylation. Cell Death Dis. 2018;9(4):423. 29555935 10.1038/s41419-018-0462-8 PMC5859188

[22] Jumper J, et al. Highly accurate protein structure prediction with AlphaFold. Nature. 2021;596(7873):583–9. 34265844 10.1038/s41586-021-03819-2 PMC8371605

[23] Abramson J, et al. Accurate structure prediction of biomolecular interactions with AlphaFold 3. Nature. 2024;630(8016):493–500. 38718835 10.1038/s41586-024-07487-w PMC11168924

[24] Kim D. et al. Integrative transcriptomic and genomic analyses unveil the IFI16 variants and expression as MASLD progression markers. Hepatology. 2025;81:962–975 10.1097/HEP.0000000000000805 38385945

[25] Shah M, et al. SARS-CoV-2 pan-variant inhibitory peptides deter S1-ACE2 interaction and neutralize delta and omicron pseudoviruses. Comput Struct Biotechnol J. 2022;20:2042–56. 35495107 10.1016/j.csbj.2022.04.030 PMC9040525

[26] Fraser TR. Lecture on the antagonism between the actions of active substances. Br Med J. 1872;2(618):485–7. 20746822 10.1136/bmj.2.618.485 PMC2295227

[27] Jenkitkasemwong S, et al. SLC39A14 is required for the development of hepatocellular iron overload in murine models of hereditary hemochromatosis. Cell Metab. 2015;22(1):138–50. 26028554 10.1016/j.cmet.2015.05.002 PMC4497937

[28] Ye Y, et al. Identification of Piwil2-like (PL2L) proteins that promote tumorigenesis. PLoS ONE. 2010;5(10):e13406. 20975993 10.1371/journal.pone.0013406 PMC2958115

[29] Houwing S, et al. A role for Piwi and piRNAs in germ cell maintenance and transposon silencing in zebrafish. Cell. 2007;129(1):69–82. 17418787 10.1016/j.cell.2007.03.026

[30] Klauer AA, van Hoof A. Degradation of mRNAs that lack a stop codon: a decade of nonstop progress. WIREs RNA. 2012;3(5):649–60. 22740367 10.1002/wrna.1124 PMC3638749

[31] Chen Y, et al. A TALEN-based specific transcript knock-down of PIWIL2 suppresses cell growth in HepG2 tumor cell. Cell Prolif. 2014;47(5):448–56. 25040173 10.1111/cpr.12120 PMC6495709

[32] Qiu B, et al. PIWIL2 stabilizes beta-catenin to promote cell cycle and proliferation in tumor cells. Biochem Biophys Res Commun. 2019;516(3):819–24. 31262447 10.1016/j.bbrc.2019.06.136

[33] Shah M, et al. The alphaC helix of TIRAP holds therapeutic potential in TLR-mediated autoimmune diseases. Biomaterials. 2020;245:119974. 32220799 10.1016/j.biomaterials.2020.119974

[34] Lu Y, et al. Cancer/testis antigen PIWIL2 suppresses circadian rhythms by regulating the stability and activity of BMAL1 and CLOCK. Oncotarget. 2017;8(33):54913–24. 28903391 10.18632/oncotarget.18973 PMC5589630

[35] Farina AK, et al. Post-transcriptional regulation of cadherin-11 expression by GSK-3 and beta-catenin in prostate and breast cancer cells. PLoS ONE. 2009;4(3):e4797. 19274078 10.1371/journal.pone.0004797 PMC2650783

[36] Gao C, Xiao G, Hu J. Regulation of Wnt/beta-catenin signaling by posttranslational modifications. Cell Biosci. 2014;4(1):13. 24594309 10.1186/2045-3701-4-13 PMC3977945

[37] Shang S, Hua F, Hu ZW. The regulation of beta-catenin activity and function in cancer: therapeutic opportunities. Oncotarget. 2017;8(20):33972–89. 28430641 10.18632/oncotarget.15687 PMC5464927

[38] Longley DB, Harkin DP, Johnston PG. 5-fluorouracil: mechanisms of action and clinical strategies. Nat Rev Cancer. 2003;3(5):330–8. 12724731 10.1038/nrc1074

[39] Ghafouri-Fard S, et al. 5-Fluorouracil: a narrative review on the role of regulatory mechanisms in driving resistance to this chemotherapeutic agent. Front Oncol. 2021;11:658636. 33954114 10.3389/fonc.2021.658636 PMC8092118

[40] Palanisamy N, et al. Rearrangements of the RAF kinase pathway in prostate cancer, gastric cancer and melanoma. Nat Med. 2010;16(7):793–8. 20526349 10.1038/nm.2166 PMC2903732

[41] Zhao C, et al. Molecular characterization and expression of Piwil1 and Piwil2 during gonadal development and treatment with HCG and LHRH-A(2) in Odontobutis potamophila. Gene. 2018;647:181–91. 29331479 10.1016/j.gene.2018.01.038

[42] Jiang S, et al. Piwil2 inhibits keratin 8 degradation through promoting p38-induced phosphorylation to resist Fas-mediated apoptosis. Mol Cell Biol. 2014;34(21):3928–38. 25113562 10.1128/MCB.00745-14 PMC4386451

[43] Mandlik DS, Mandlik SK, Choudhary HB. Immunotherapy for hepatocellular carcinoma: current status and future perspectives. World J Gastroenterol. 2023;29(6):1054–75. 36844141 10.3748/wjg.v29.i6.1054 PMC9950866

[44] Zuo ZH, et al. Oncogenic activity of solute carrier family 45 member 2 and alpha-methylacyl-coenzyme A racemase gene fusion is mediated by mitogen-activated protein kinase. Hepatol Commun. 2022;6(1):209–22. 34505419 10.1002/hep4.1724 PMC8710797

