# Correction: Peptide‑based therapeutics targeting the SLC39A14‑PIWIL2 fusion in hepatocellular carcinoma

**DOI:** 10.1186/s44342-025-00066-z

**Published:** 2026-04-16

**Authors:** Masaud Shah, Sung Ung Moon, Ji‑Hye Choi, Min Jae Kim, Hyun Goo Woo

**Affiliations:** 1 Department of Physiology, Ajou University School of Medicine, Suwon, 16499 Republic of Korea; 2 Department of Biomedical Science, Graduate School, Ajou University, Suwon, 16499 Republic of Korea; 3 Department of Pathology, Yonsei University College of Medicine, Seoul, Republic of Korea; 4 Ajou Translational Omics Center (ATOC), Research Institute for Innovative Medicine, Ajou University Medical Center, Suwon, Republic of Korea

**Correction: Genom Inform 23, 28 (2025)**

**https://doi.org/10.1186/s44342-025-00060-5**

Following publication of the original article [1], the authors identified an error in the competing interests section.

The incorrect competing interests section reads:

**Competing interests**

The authors declare no competing interests.

The correct competing interests section reads:

**Competing interests**

Author Hyun Goo Woo is Editor-in-Chief of *Genomics & Informatics*. The paper was handled by another Editor and underwent a rigorous peer review process. Author Hyun Goo Woo was not involved in the journal's peer review of, or decisions related to, this manuscript. In addition, author Masaud Shah is also a member of the editorial board of *Genomics & Informatics* and was not involved in the journal's peer review of, or decisions related to, this manuscript. The remaining authors declare no competing interests. All data and subsequent analyses were conducted without the use of AI tools, except for the AlphaFold 3 server, which was used for protein model.

The competing interests section has been updated above and the original article [1] has been corrected.
