# Supplementary material for: Peptide‑based therapeutics targeting the SLC39A14‑PIWIL2 fusion in hepatocellular carcinoma
Source: Genomics Inform. 2025 Dec 20;23:28. doi: 10.1186/s44342-025-00060-5 (PMC12720462; doi:10.1186/s44342-025-00060-5)
Supplement: Supplementary file 1 — Supplementary Material 1. [file 44342_2025_60_MOESM1_ESM.pdf]

HPLC REPORT

**Product Name**	FONB1 Peptide Synthesis			
**Catalog Number**	CSB-DT1054			
**Sequence**	*RQIKIWFQNRRMKWKKVVQSPVPSQVVNV*			
**Lot No**	M0906P11			
**Column**	Gemini-NX 5 μ C18 110A, 4.6*250mm			
**Solvent A**	0.1% Trifluoroacetic in 100% Acetonitrile			
**Solvent B**	0.1% Trifluoroacetic in 100% Water			
**Purification**	Affinity purified using IMAC			
**Gradient**		A	B	
	0.01 min	25%	75%	
	25 min	50%	50%	
	25.1 min	100%	0%	
	30 min	STOP		
**Flow rate**	1.0 mL/min			
**Wavelength**	220nm			
**Volume**	20ul			
**Peak No.**	**Ret Time**	**Height**	**Area**	**Conc.**
**1**	8.003	19242	111626	1.2525
**2**	8.176	1206279	8519056	95.5849
**3**	8.527	31023	281867	3.1626
**Total**		1256544	8912549	100
**Purity**	95.58%			
**Suggested Solvent (or solvent used in purification)**	1.0 mg peptide dissolve with 1ml DMSO			
**Form**	Lyophilized powder			
**Manufacture Date**	2023-9-6			

**Recommended Storage**	Short term	4 ℃, one week from the date of receipt
	Long term	-20 to -80 ℃, 6 months from the date of receipt
**Notes**	The shelf life is related to many factors, storage state, storage temperature and the stability of the peptide itself. The above shelf lives are concluded by our current storage experience. The specific shelf life will depend on specific peptide.<br>Please use as soon as possible after receiving. Repeated freezing and thawing is not recommended.	

MASS SPECTROMETRY REPORT

**MASS Sample Description**	
**Analyzed date**	2023-9-6
**Analyst**	Shen
**Sample**	CSB-DT1054
**M.W.**	3580.25Da
**Lot. No.**	M0906P11
**Instrument**	Agilent-6125B
**Probe**	ESI
**Nebulizer Gas Flow**	1.5L/min
**CDL**	-20.0v
**CDL Temp.**	250°C
**Block Temp.**	200°C
**Probe Bias**	+ 4.5kv
**Detector**	1.5kv
**T. Flow**	0.2ml/min
**B. Conc.**	50%H_2O/50%ACN

MASS

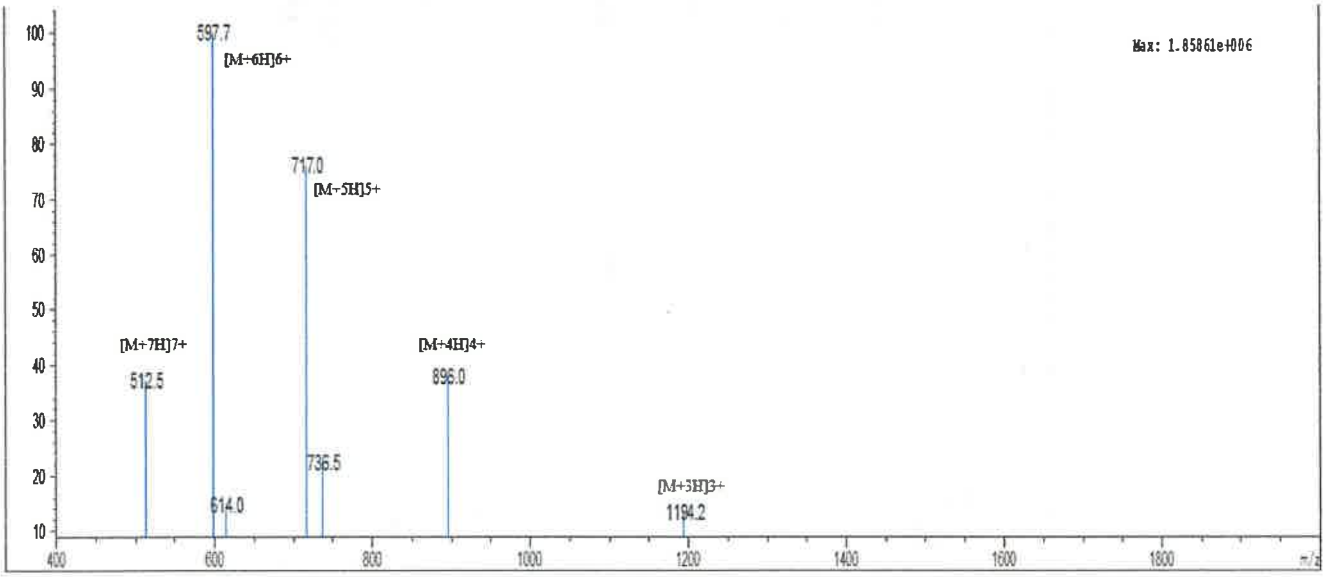
597.7
[M+6H]6+
717.0
[M+5H]5+
[M+7H]7+
512.5
[M+4H]4+
896.0
736.5
614.0
[M+3H]3+
1194.2
Max: 1.85861e+006
100
90
80
70
60
50
40
30
20
10
400
600
800
1000
1200
1400
1600
1800
m/z

HPLC

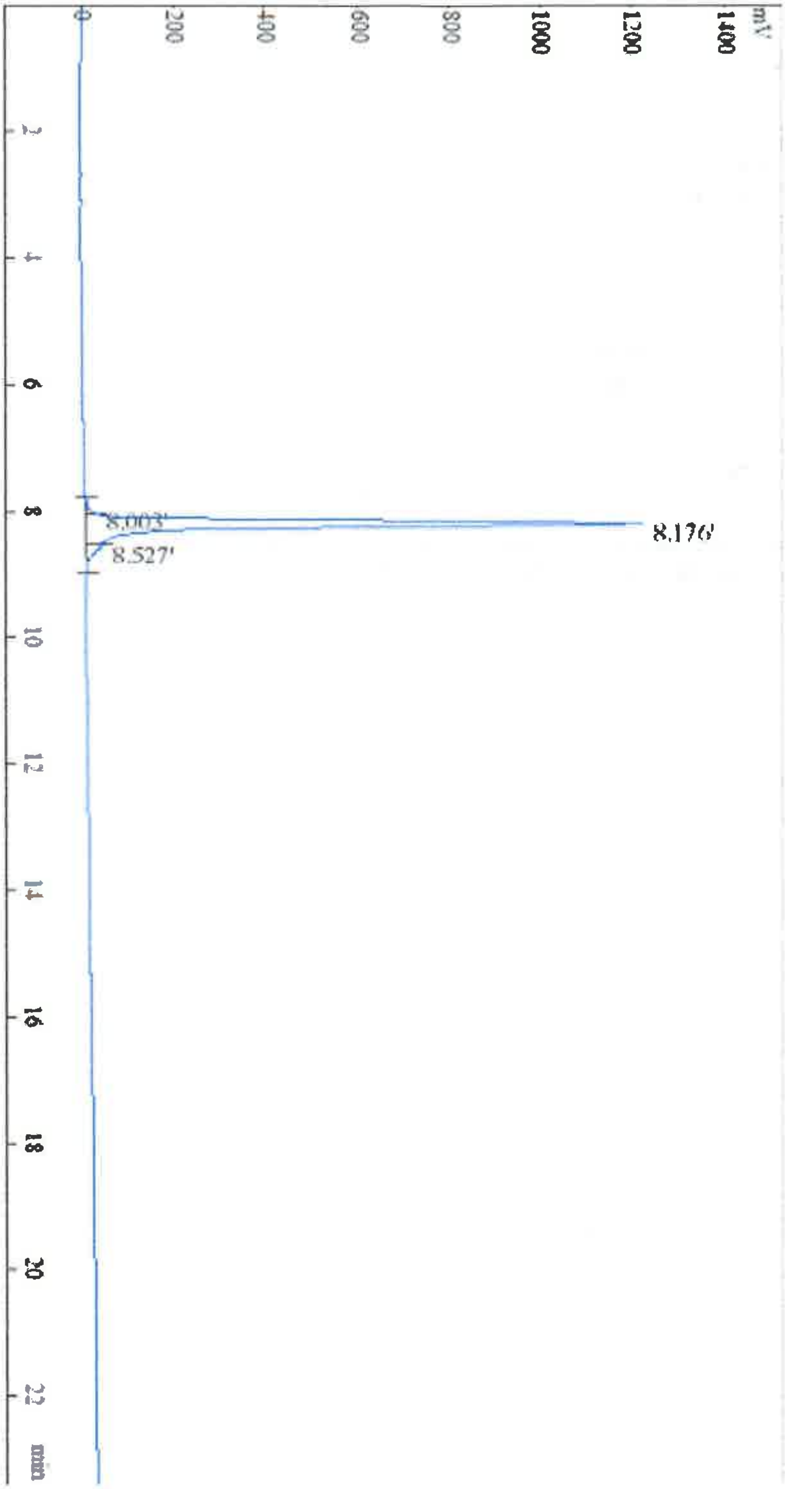
mV
0
200
400
600
800
1000
1200
1400
min
2
4
6
8
10
12
14
16
18
20
22
8.003'
8.176'
8.527'

HPLC REPORT

**Product Name**	FONB2 Peptide Synthesis			
**Catalog Number**	CSB-DT1055			
**Sequence**	*RQIKIWFQNRRMKWKKQEIVDSLKLSLVGSLKKFYEVN*			
**Lot No**	M0920P2			
**Column**	Gemini-NX 5 μ C18 110A, 4.6*250mm			
**Solvent A**	0.1% Trifluoroacetic in 100% Acetonitrile			
**Solvent B**	0.1% Trifluoroacetic in 100% Water			
**Purification**	Affinity purified using IMAC			
**Gradient**		A	B	
	0.01 min	20%	80%	
	25 min	80%	20%	
	25.1 min	100%	0%	
	30 min	STOP		
**Flow rate**	1.0 mL/min			
**Wavelength**	220nm			
**Volume**	20ul			
**Peak No.**	**Ret Time**	**Height**	**Area**	**Conc.**
1	11.251	2393	46737	0.6557
2	11.852	6938	152700	2.1424
3	12.206	433456	6872658	96.4265
4	12.718	2101	55268	0.7754
**Total**		444888	7127363	100
**Purity**	96.43%			
**Suggested Solvent (or solvent used in purification)**	1.0 mg peptide dissolve with 1ml DMSO			
**Form**	Lyophilized powder			
**Manufacture Date**	2023-9-20			

**Recommended Storage**	Short term	4 ℃, one week from the date of receipt
	Long term	-20 to -80 ℃, 6 months from the date of receipt
**Notes**	The shelf life is related to many factors, storage state, storage temperature and the stability of the peptide itself. The above shelf lives are concluded by our current storage experience. The specific shelf life will depend on specific peptide.<br>Please use as soon as possible after receiving. Repeated freezing and thawing is not recommended.	

MASS SPECTROMETRY REPORT

MASS Sample Description	
**Analyzed date**	2023-9-20
**Analyst**	Shen
**Sample**	CSB-DT1055
**M.W.**	4738.6Da
**Lot. No.**	M0920P2
**Instrument**	Agilent-6125B
**Probe**	ESI
**Nebulizer Gas Flow**	1.5L/min
**CDL**	-20.0v
**CDL Temp.**	250℃
**Block Temp.**	200℃
**Probe Bias**	+ 4.5kv
**Detector**	1.5kv
**T. Flow**	0.2ml/min
**B. Conc.**	50%H_2O/50%ACN

HPLC

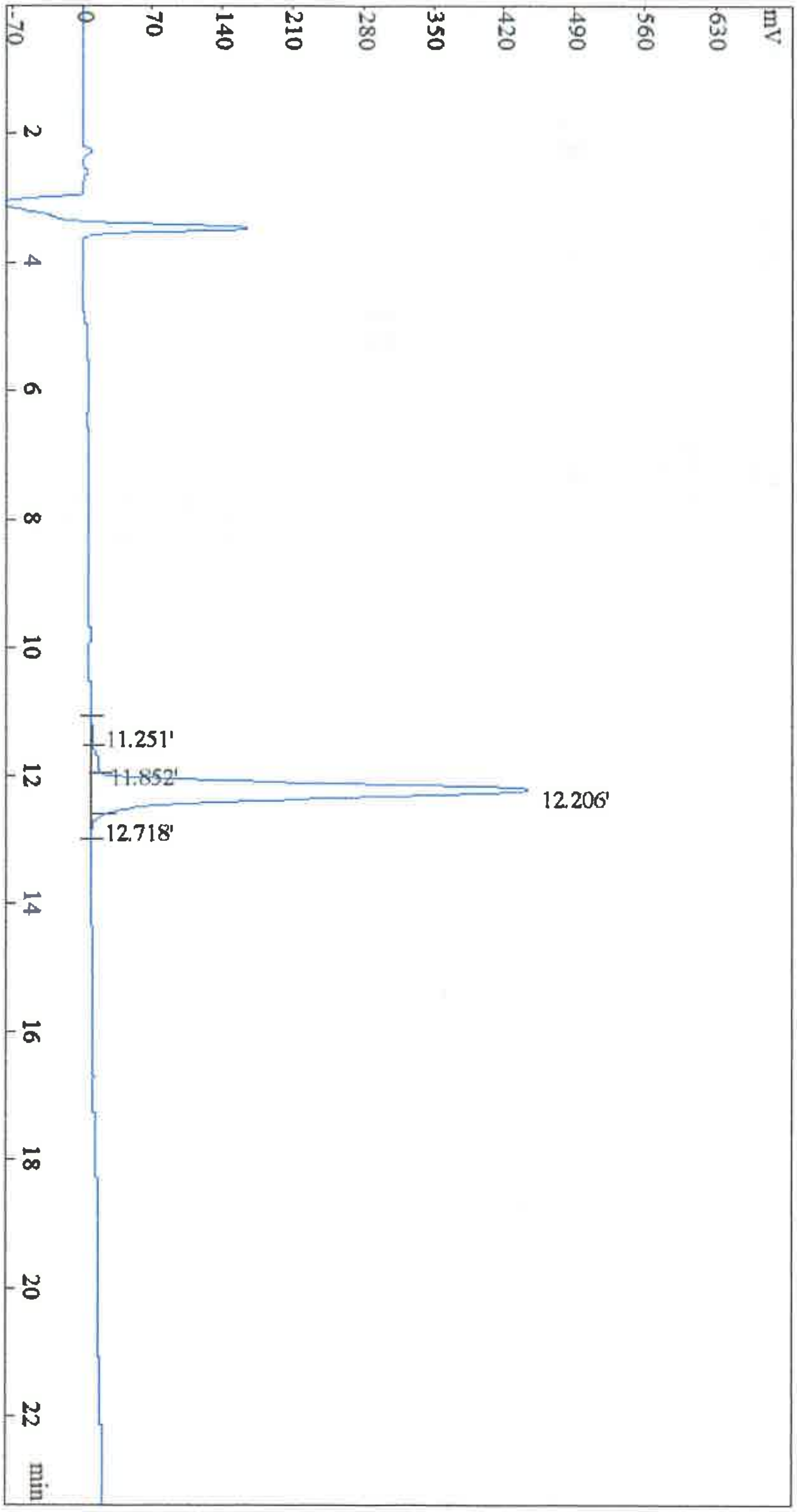
mV
-70
0
70
140
210
280
350
420
490
560
630
min
2
4
6
8
10
12
14
16
18
20
22
11.251'
11.852'
12.206'
12.718'

MASS

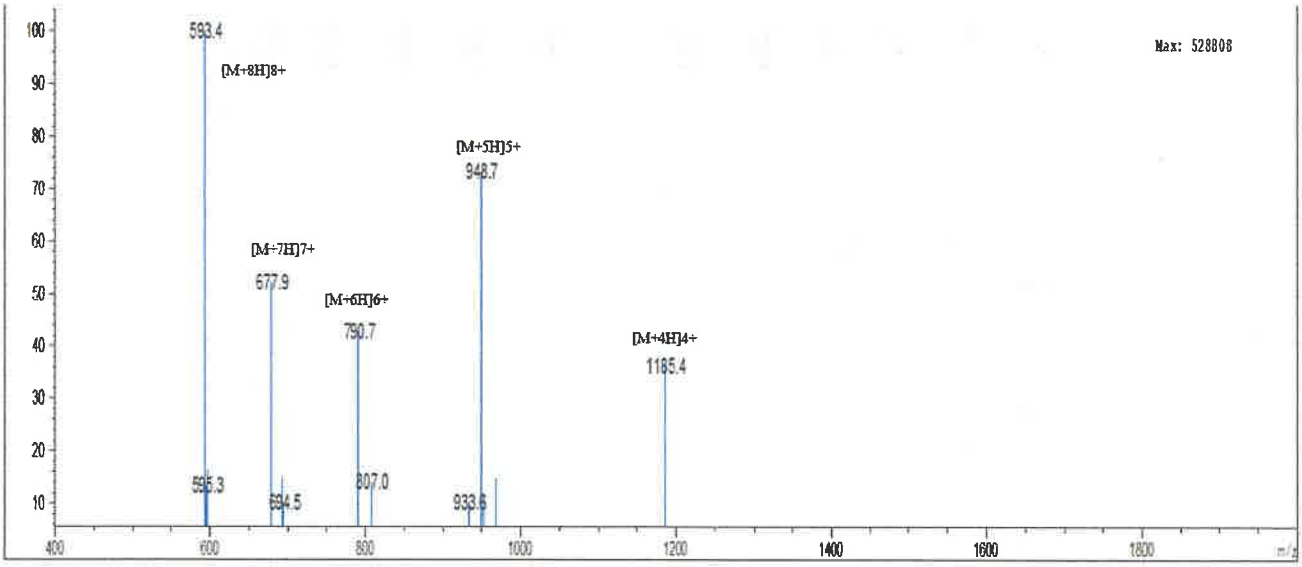
Max: 528808
593.4
[M+8H]8+
[M+7H]7+
677.9
[M+6H]6+
790.7
[M+5H]5+
948.7
[M+4H]4+
1185.4
595.3
694.5
807.0
933.6
100
90
80
70
60
50
40
30
20
10
400
600
800
1000
1200
1400
1600
1800
m/z

CUSABIO®
CUSABIO TECHNOLOGY LLC
Tel:+1-301-363-4651 Email:cusabio@cusabio.com Website:www.cusabio.com

HPLC REPORT

**Product Name**	Nep1 Peptide Synthesis			
**Catalog Number**	CSB-DT1059			
**Sequence**	*RQIKIWFQNRRMKWKKRLVAMKFLRASEEHLKQHYIDLK*			
**Lot No**	M0913P1			
**Column**	Gemini-NX 5 μ C18 110A, 4.6*250mm			
**Solvent A**	0.1% Trifluoroacetic in 100% Acetonitrile			
**Solvent B**	0.1% Trifluoroacetic in 100% Water			
**Purification**	Affinity purified using IMAC			
**Gradient**		A	B	
	0.01 min	25%	75%	
	25 min	50%	50%	
	25.1 min	100%	0%	
	30 min	STOP		
**Flow rate**	1.0 mL/min			
**Wavelength**	220nm			
**Volume**	20ul			
**Peak No.**	**Ret Time**	**Height**	**Area**	**Conc.**
**1**	9.303	391990	4162116	96.9451
**2**	9.783	15065	131155	3.0549
**Total**		407055	4293271	100
**Purity**	96.95%			
**Suggested Solvent (or solvent used in purification)**	1.0 mg peptide dissolve with 1ml DMSO			
**Form**	Lyophilized powder			
**Manufacture Date**	2023-9-14			
**Recommended Storage**	Short term	4 ℃, one week from the date of receipt		
	Long term	-20 to -80 ℃, 6 months from the date of receipt		

**Notes**	The shelf life is related to many factors, storage state, storage temperature and the stability of the peptide itself. The above shelf lives are concluded by our current storage experience. The specific shelf life will depend on specific peptide.<br>Please use as soon as possible after receiving. Repeated freezing and thawing is not recommended.

MASS SPECTROMETRY REPORT

**MASS Sample Description**	
**Analyzed date**	2023-9-14
**Analyst**	Shen
**Sample**	CSB-DT1059
**M.W.**	5055.03Da
**Lot. No.**	M0913P1
**Instrument**	Agilent-6125B
**Probe**	ESI
**Nebulizer Gas Flow**	1.5L/min
**CDL**	-20.0v
**CDL Temp.**	250℃
**Block Temp.**	200℃
**Probe Bias**	+ 4.5kv
**Detector**	1.5kv
**T. Flow**	0.2ml/min
**B. Conc.**	50%H_2O/50%ACN

MASS

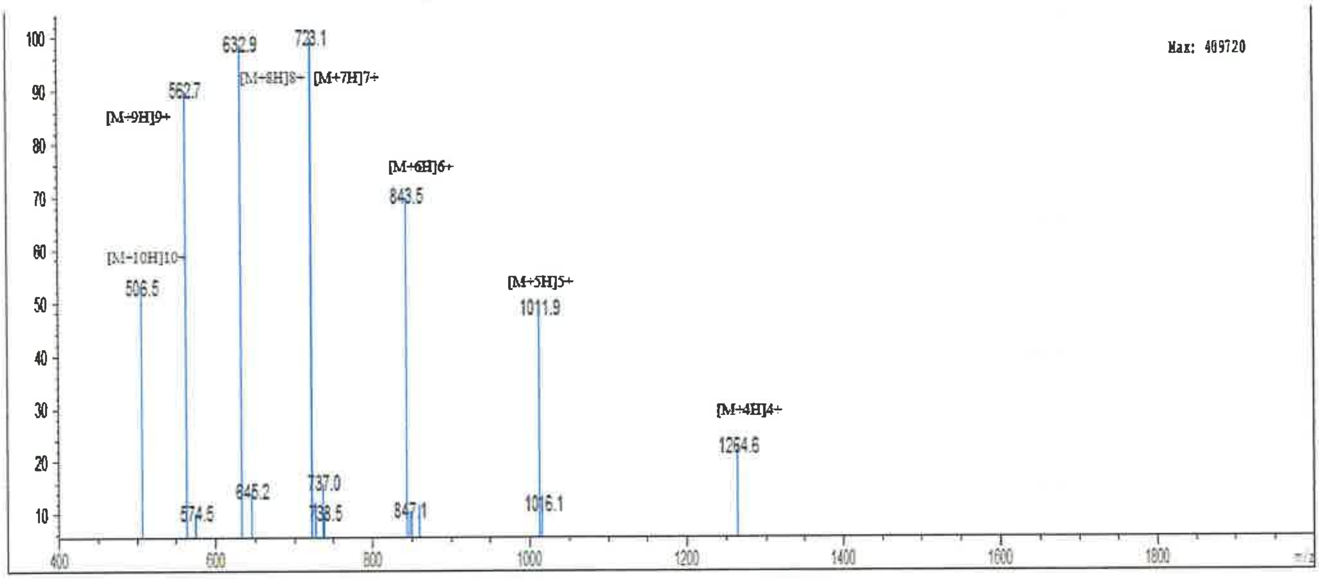
Max: 409720
632.9
723.1
562.7
[M+8H]8+
[M+7H]7+
[M+9H]9+
[M+6H]6+
843.5
[M+10H]10+
506.5
[M+5H]5+
1011.9
[M+4H]4+
1264.6
645.2
737.0
574.5
738.5
847.1
1016.1
100
90
80
70
60
50
40
30
20
10
400
600
800
1000
1200
1400
1600
1800

HPLC

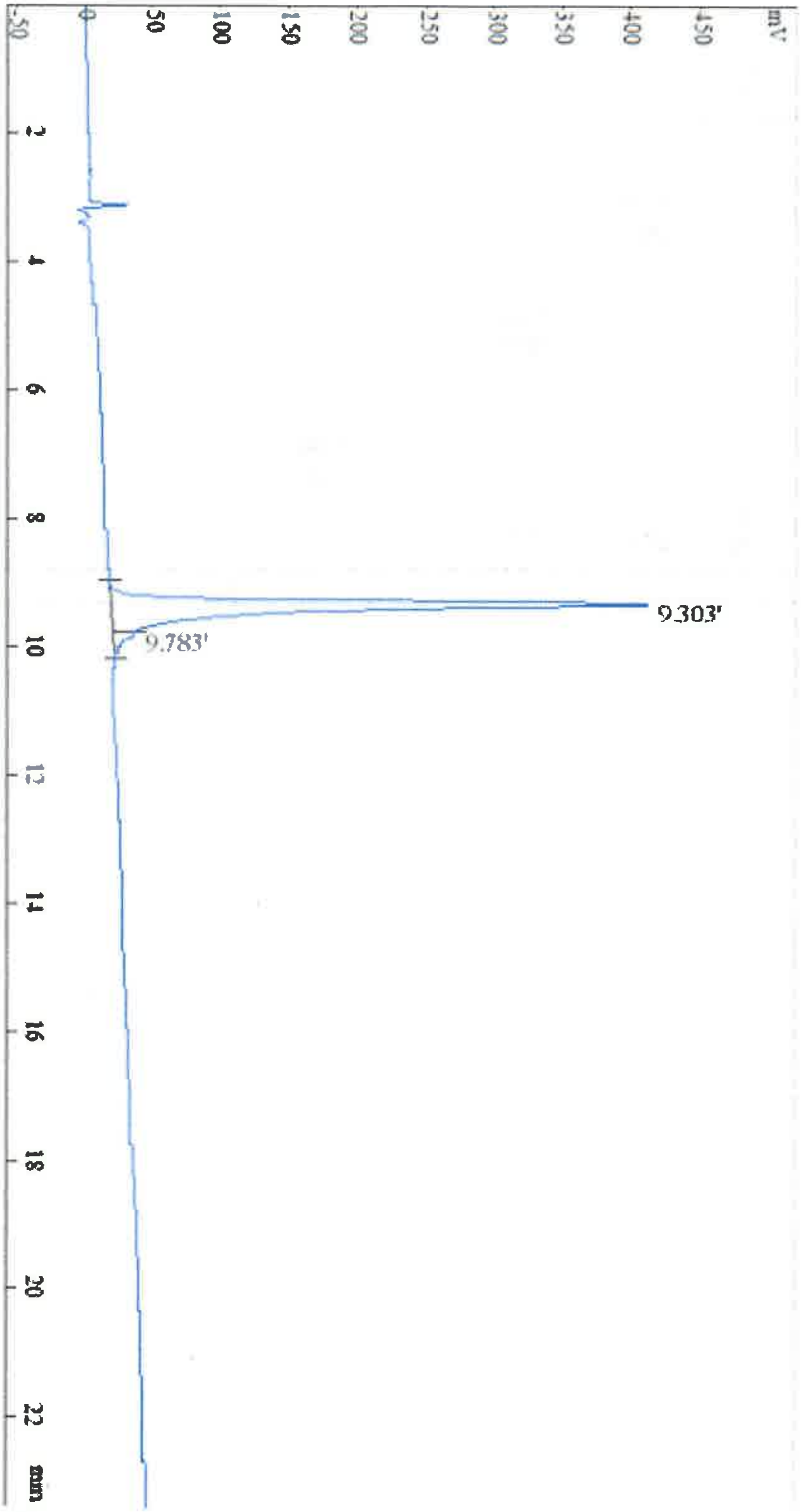

CUSABIO

CUSABIO TECHNOLOGY LLC

Tel:+1-301-363-4651 Email:cusabio@cusabio.com Website:www.cusabio.com

HPLC REPORT

**Product Name**	Hdep1 Peptide Synthesis			
**Catalog Number**	CSB-DT1057			
**Sequence**	*RQIKIWFQNRRMKWKKSRQYLDQIRQTIFENLKMLN*			
**Lot No**	M0926P1			
**Column**	Gemini-NX 5 μ C18 110A, 4.6*250mm			
**Solvent A**	0.1% Trifluoroacetic in 100% Acetonitrile			
**Solvent B**	0.1% Trifluoroacetic in 100% Water			
**Purification**	Affinity purified using IMAC			
**Gradient**		A	B	
	0.01 min	20%	80%	
	25 min	80%	20%	
	25.1 min	100%	0%	
	30 min	STOP		
**Flow rate**	1.0 mL/min			
**Wavelength**	220nm			
**Volume**	20ul			
**Peak No.**	**Ret Time**	**Height**	**Area**	**Conc.**
**1**	12.425	10481	63413	0.6557
**2**	12.734	586947	9257890	95.7351
**3**	13.226	21514	349020	3.6092
**Total**		618942	9670323	100
**Purity**	95.74%			
**Suggested Solvent (or solvent used in purification)**	1.0 mg peptide dissolve with 1ml DMSO			
**Form**	Lyophilized powder			
**Manufacture Date**	2023-9-27			
**Recommended**	Short term	4 ℃, one week from the date of receipt		

**Storage**	Long term	-20 to -80 ℃, 6 months from the date of receipt
**Notes**	The shelf life is related to many factors, storage state, storage temperature and the stability of the peptide itself. The above shelf lives are concluded by our current storage experience. The specific shelf life will depend on specific peptide.<br>Please use as soon as possible after receiving. Repeated freezing and thawing is not recommended.	

MASS SPECTROMETRY REPORT

MASS Sample Description	
**Analyzed date**	2023-9-27
**Analyst**	Shen
**Sample**	CSB-DT1057
**M.W.**	4739.57Da
**Lot. No.**	M0926P1
**Instrument**	Agilent-6125B
**Probe**	ESI
**Nebulizer Gas Flow**	1.5L/min
**CDL**	-20.0v
**CDL Temp.**	250℃
**Block Temp.**	200℃
**Probe Bias**	+ 4.5kv
**Detector**	1.5kv
**T. Flow**	0.2ml/min
**B. Conc.**	50%H_2O/50%ACN

HPLC

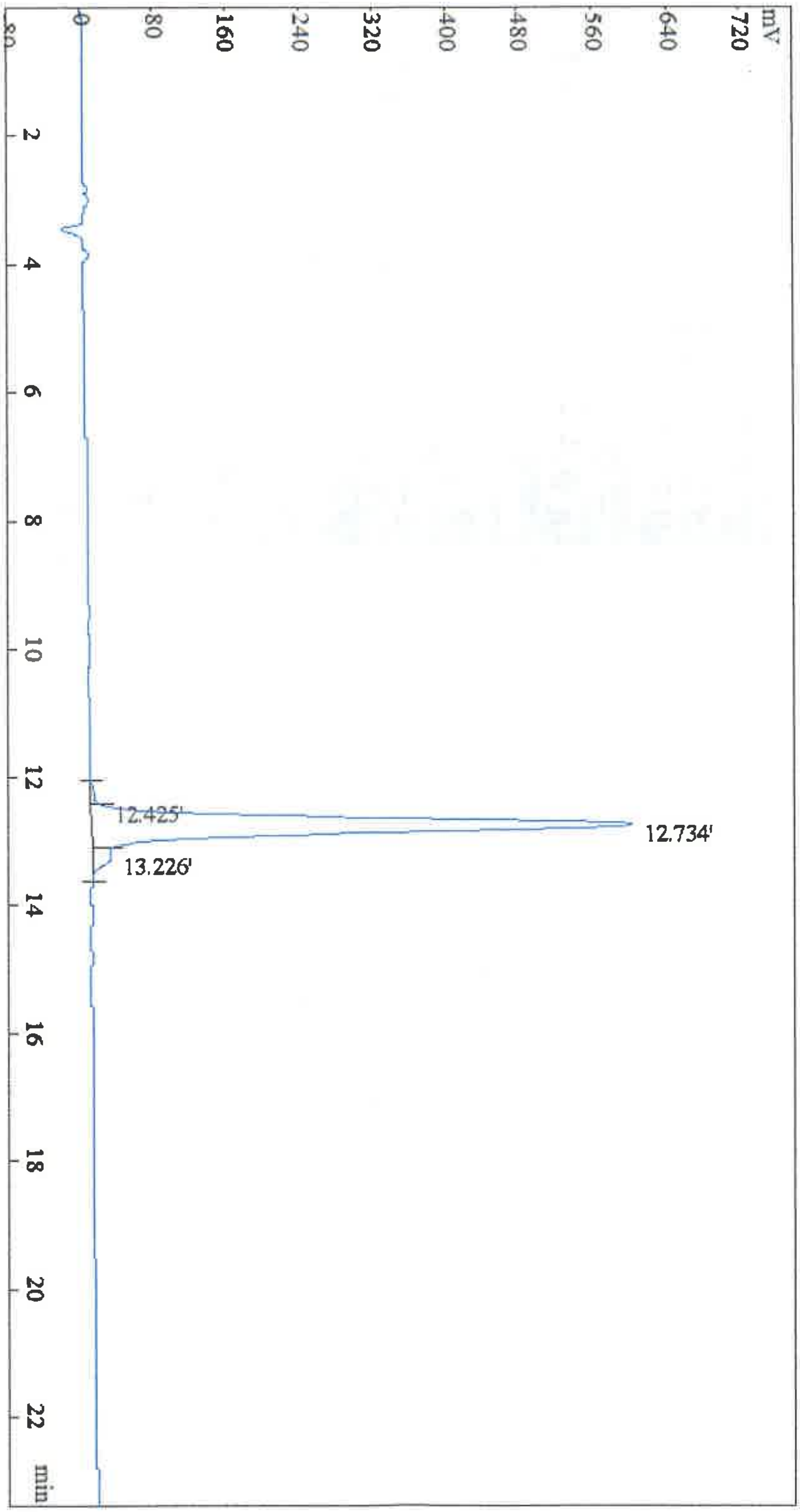
mV
0
80
160
240
320
400
480
560
640
720
2
4
6
8
10
12
14
16
18
20
22
min
12.425'
12.734'
13.226'

MASS

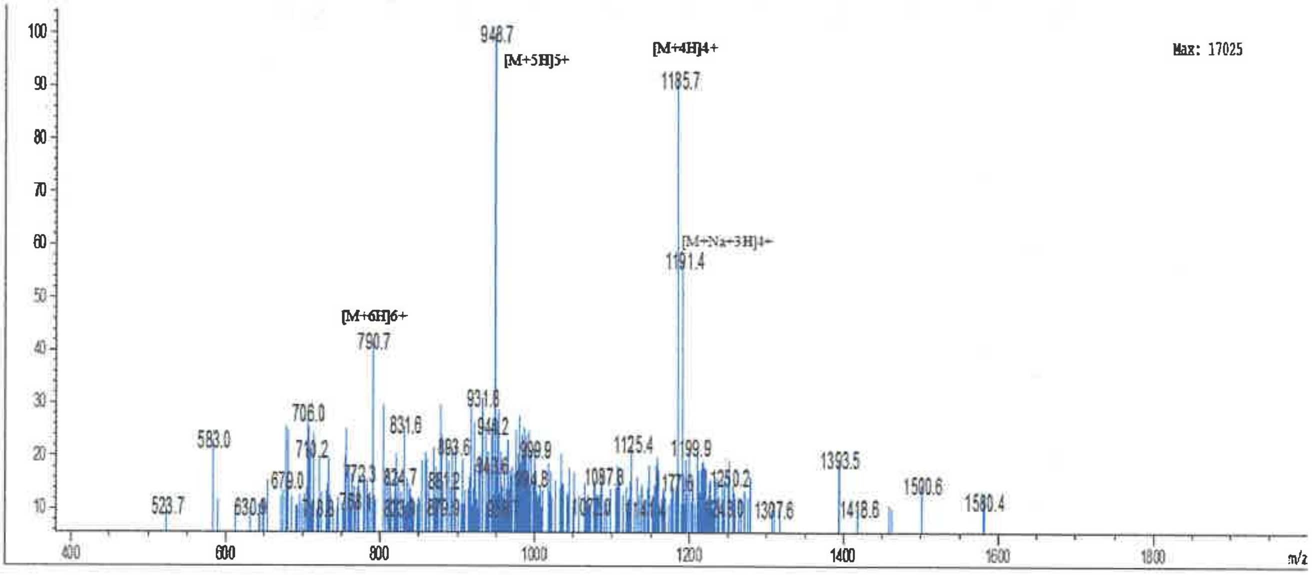
Max: 17025
948.7
[M+5H]5+
[M+4H]4+
1185.7
[M+Na+3H]4+
1191.4
[M+6H]6+
790.7
931.8
706.0
831.6
583.0
1125.4
1199.9
1393.5
1500.6
1580.4
523.7
1307.6
1418.6
679.0
772.3
824.7
1087.8
1250.2
m/z
400
600
800
1000
1200
1400
1600
1800
100
90
80
70
60
50
40
30
20
10
