# Supplementary material for: Peptide‑based therapeutics targeting the SLC39A14‑PIWIL2 fusion in hepatocellular carcinoma
Source: Genomics Inform. 2025 Dec 20;23:28. doi: 10.1186/s44342-025-00060-5 (PMC12720462; doi:10.1186/s44342-025-00060-5)

**Peptide‑Based Therapeutics Targeting the SLC39A14‑PIWIL2 Fusion in Hepatocellular Carcinoma**

Masaud Shah*^1+^*, Sung Ung Moon*^1+^*, Ji-Hye Choi*^1,2+^*, Min Jae Kim*^2^*, and Hyun Goo Woo*^1,2,3^**

^1^Department of Physiology, Ajou University School of Medicine, Suwon 16499, Republic of Korea. ^2^Department of Biomedical Science, Graduate School, Ajou University, Suwon 16499, Republic of Korea. ^3^Department of Pathology, Yonsei University College of Medicine, Seoul, Republic of Korea. ^3^Ajou Translational Omics Center (ATOC), Research Institute for Innovative Medicine, Ajou University Medical Center, Suwon, Republic of Korea.

**Table S1. Reverse transcription quantitative PCR primers.**

| **Gene** | **5` primers** | **3` primers** |
| --- | --- | --- |
| **PIWIL2** | **5`-TGAAGAAGGACTTCAGAGCCATG-3’** | **5`-CGCATCAGTTCATTGGTGGCTG-3’** |
| **CTNNB1** | **5`-CACAAGCAGAGTGCTGAAGGTG-3’** | **5`-GATTCCTGAGAGTCCAAAGACAG-3`** |
| **c-MYC** | **5`-CCTGGTGCTCCATGAGGAGAC-3`** | **5`-CAGACTCTGACCTTTTGCCAGG-3`** |
| **TP53** | **5`-CCTCAGCATCTTATCCGAGTGG-3’** | **5`-TGGATGGTGGTACAGTCAGAGC-3’** |
| **Cyclin D1** | **5`-TCTACACCGACAACTCCATCCG-3’** | **5`-TCTGGCATTTTGGAGAGGAAGTG-3’** |
| **hNME2** | **5`-AGGATTCCGCCTTGTTGGTCTG-3’** | **5`-CGGCAAAGAATGGACGGTCCTT-3’** |
| **GAPDH** | **5`-GTCTCCTCTGACTTCAACAGCG-3`** | **5`-ACCACCCTGTTGCTGTAGCCAA-3`** |
| **SLC39A14_PIWIL2_fusion** | **5`- CACCGGCTAAGCTGCTTCTG -3`** | **5`- GTTGCCGGTGACAGCTTGAT -3`** |
| **Predictive_promoter_region_Set_1** | **5`- CACTGTAATTACGGGGTGAGGA -3`** | **5`- GCAGCTCCTGAAAAGCAAAG -3`** |
| **Predictive_promoter_region_Set_2-3** | **5`- GTAATATGAAGAATCTGACCCCCTC -3`** | **5`- GCGGCAGAAGCAGCTTAG -3`** |
| **WT_tPIWIL2_PCDNA3** | **5`-CAGTGTGCTGGAATTCATGGATCCTTTCCGACCATCG -3`** | **5`-ATAGGGCCCTCTAGATCACAGGAAGAACAGGTTCTCGC-3`** |
| **SLC39A14_PCDNA3_F and**  **Truncated_PIWIL2_PCDNA3_F** | **5`- CAGTGTGCTGGAATTCATGATATAGCCGGGCATGCGC -3`** | **5`-CAGTGTGCTGGAATTCATGAGGTTCGGCATGTTGAAGG-3`** |

**Figure S1. Comparison of the experimental and modeled structure of PIWIL2 and its domains.**  Structural modeling and stability evaluation of the PIWIL2 protein. The truncated PIWIL2 protein was modeled using AlphaFold 3 and superimposed onto the Cryo-EM structure (blue cartoon). The overall RMSD was calculated as 1.46 Å.


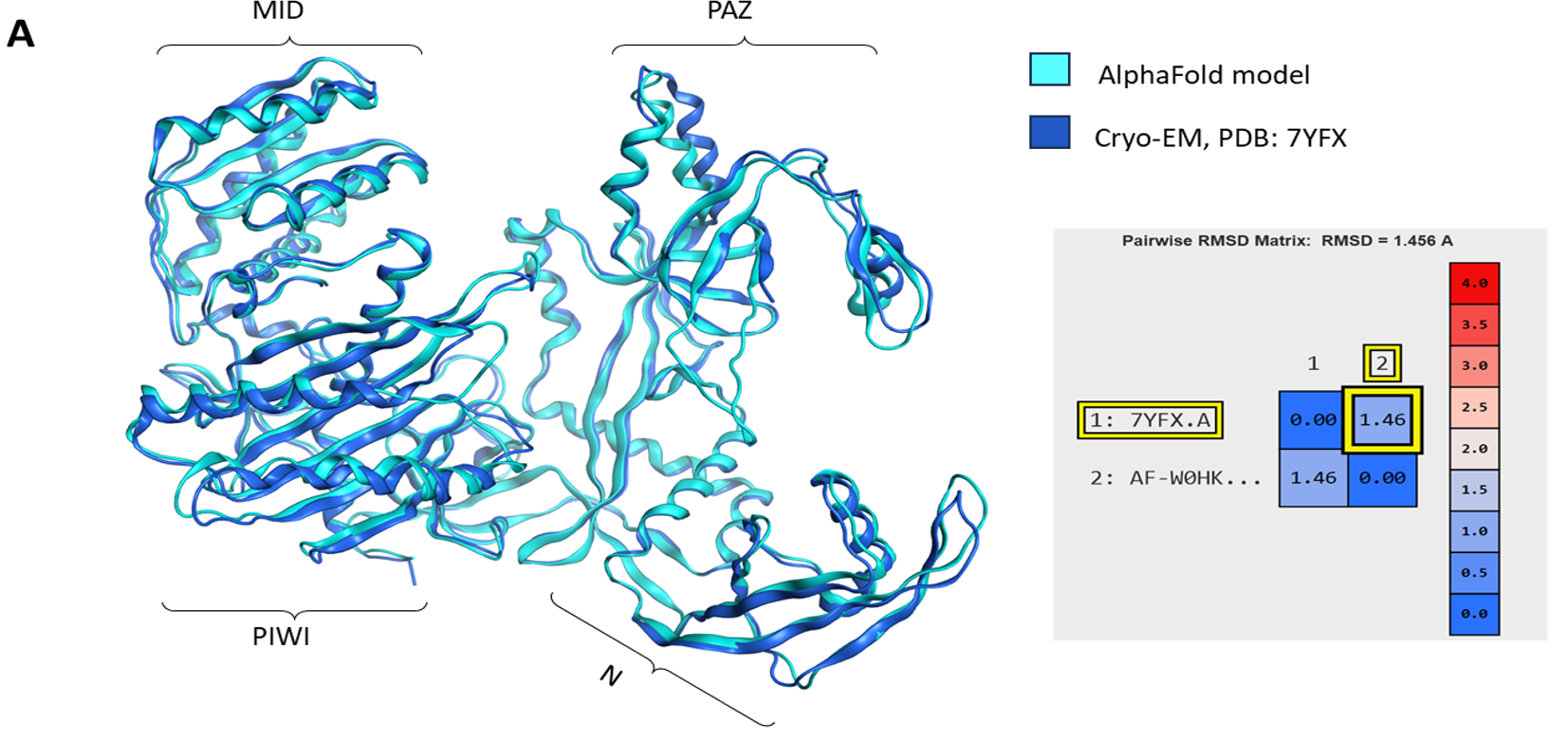


**Figure S2. Characterization of the SLC39A14-PIWIL2 Fusion Gene and Its Expression Across Cancer Types. (A)** The nucleotide sequence of the SLC39A14-PIWIL2 fusion transcript was translated using Expasy-translate. The green-highlighted region indicates the translated sequence. The fusion results in the exclusion of exon 1 from SLC39A14, producing a truncated PIWIL2 protein lacking exons 1–6. **(B)** RNA expression levels of SLC39A14 and PIWIL2 in HCC compared to control samples. **(C)** Identification of SLC39A14-PIWIL2 fusion products in STAD and LUSC cancers, revealing distinct breakpoints and potential functional variations.


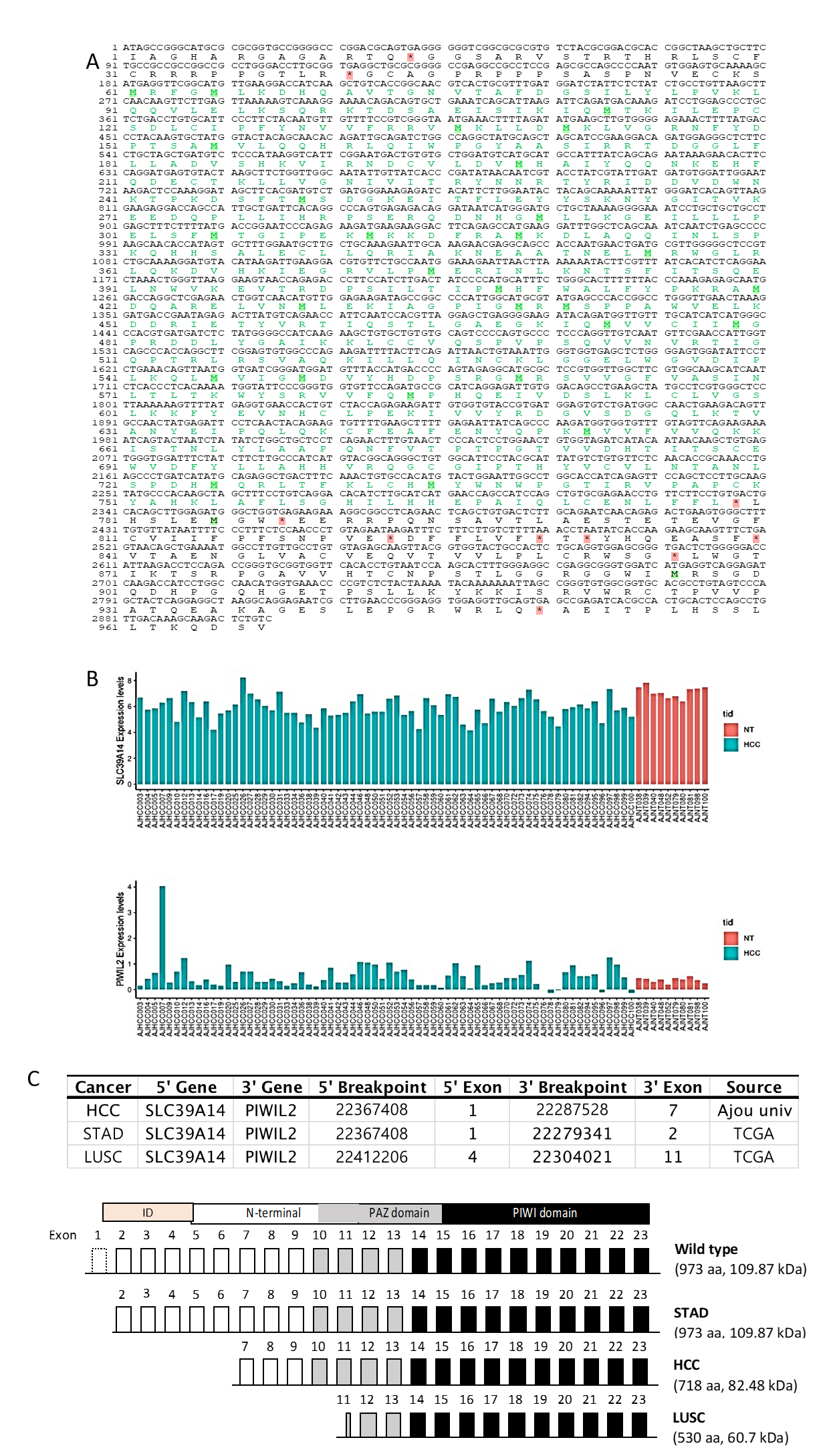


**Figure S3. Expression Profiles of SLC39A14 and PIWIL2 in Human Tissues and HCC Cell Lines. (A)** Expression of SLC39A14 across various human organs and tissues, as indicated by the Human Protein Atlas database. **(B)** Expression of PIWIL2 across different human organs and tissues, as indicated by the Human Protein Atlas database. **(C)** Expression of the SLC39A14 gene under its predicted promoter. **(D)** Relative RNA abundance of SLC39A14-PIWIL2 fusion products in human HCC cell lines, Huh7 and HepG2, upon their respective plasmid transfection.


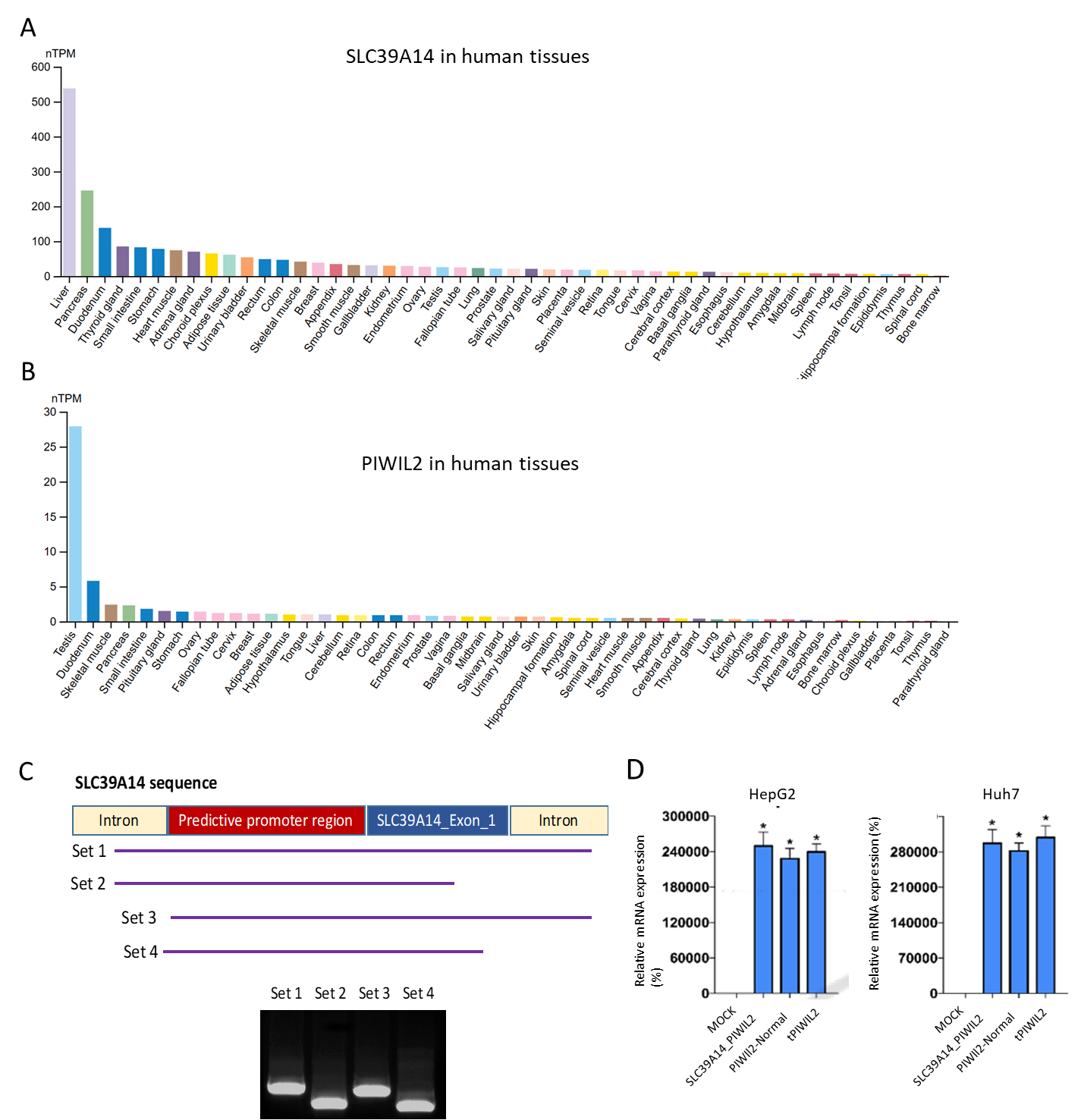


**Figure S4. Optimization of PIWIL2-overexpressing liver cancer cell lines, assessment of phenotypic changes, and evaluation of peptide toxicity.** (A) RT-qPCR analysis was performed to measure changes in PIWIL2 mRNA expression following PIWIL2 overexpression (PIWIL2oe) in Huh7, HepG2, SNU398, and SNU449 cells. Relative mRNA levels of target genes were normalized to GAPDH. (B) Cell viability was assessed using the CellTiter-Blue assay (555Ex/585Em) in liver cancer cell lines (Huh7, HepG2, SNU398, and SNU449) after treatment with either the PIWIL2oe plasmid or the vector control at seeding densities of 2,000, 4,000, 8,000, and 10,000 cells. (C) Sphere formation was analyzed in ultra-low attachment plates for vector and PIWIL2oe liver cancer cells (Huh7, HepG2, SNU398, and SNU449) seeded at 2,000, 4,000, 8,000, and 10,000 cells. Sphere formation was evaluated using cell viability analysis and presented in the bar chart. The upper panel shows images of sphere formation, while the lower panel depicts loose cell viability. Values represent the mean ± standard deviation of three independent experiments. (D) The toxicity of the NEP1 peptide was evaluated in the normal cell line HEK293 using cell viability analysis at varying seeding densities or NEP1 concentrations. Statistical significance is indicated as *P < 0.05, **P < 0.01, and ***P < 0.001 vs. vector control.


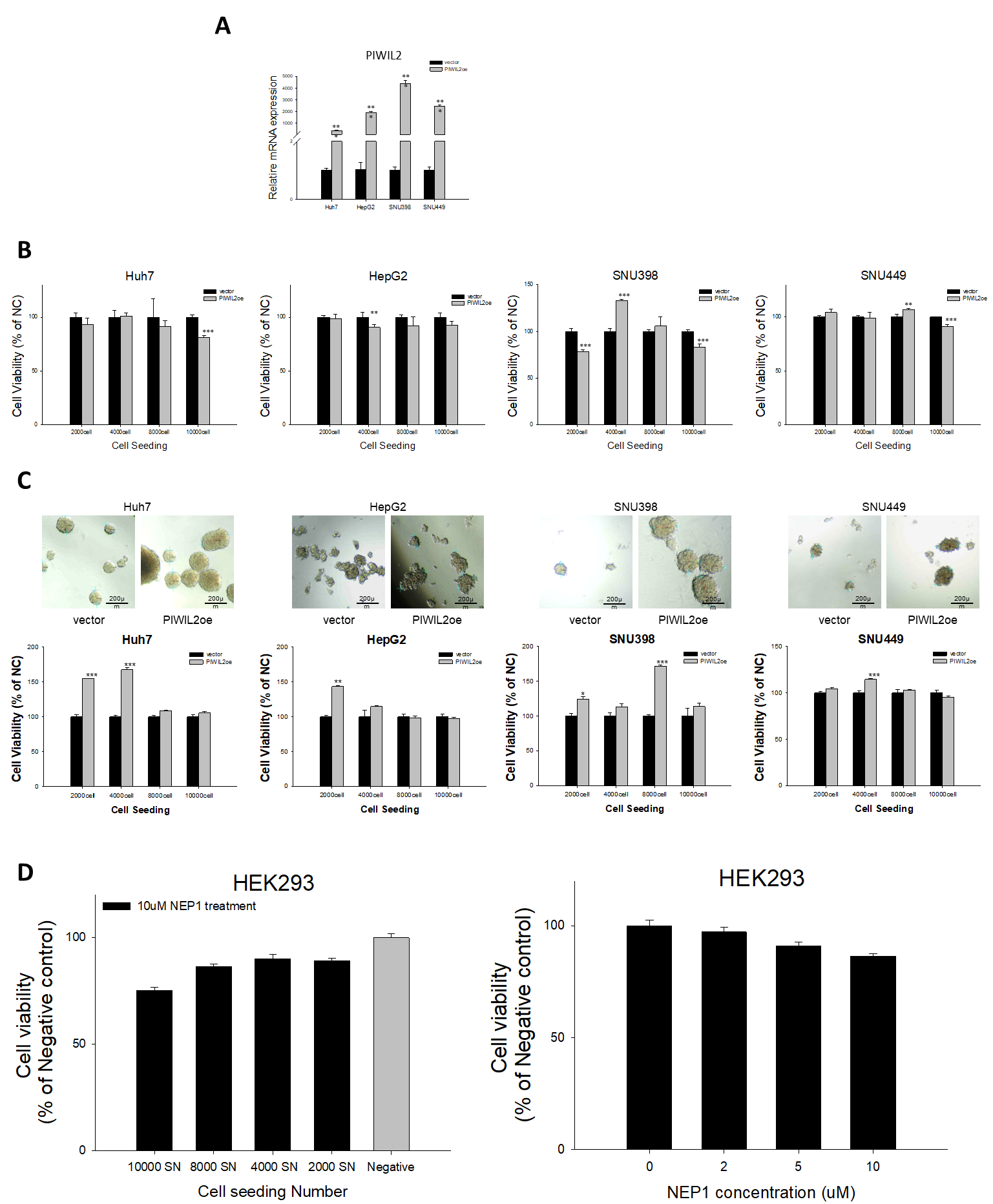


**Raw Western Blot pictures**

c-MYC-SNU398


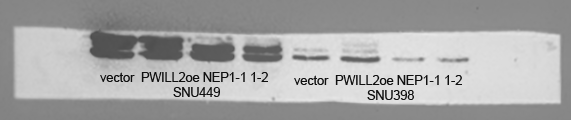


c-MYC-SNU449


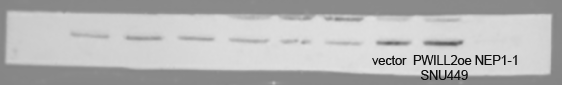


CTNNB1


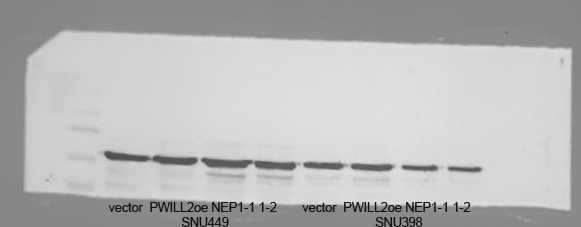


pAKT-SNU398


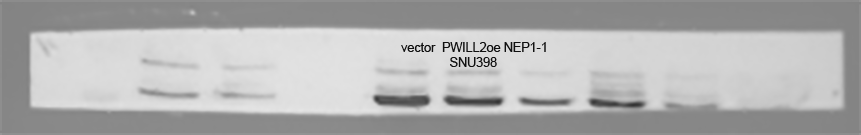


pAKT-SNU449


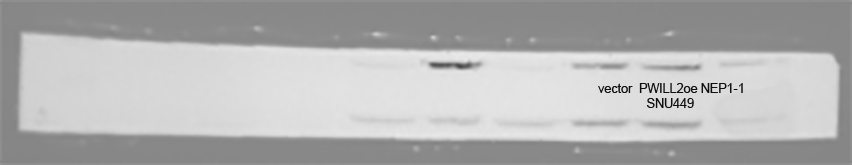


pGSK3β-SNU398


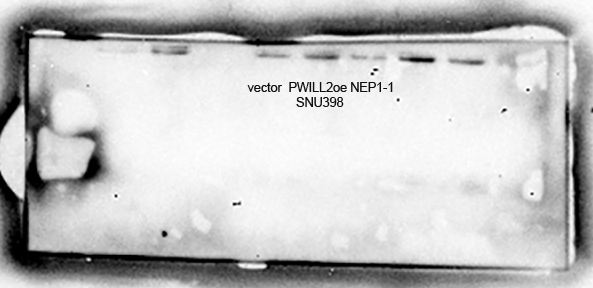


pGSK3β-SNU449


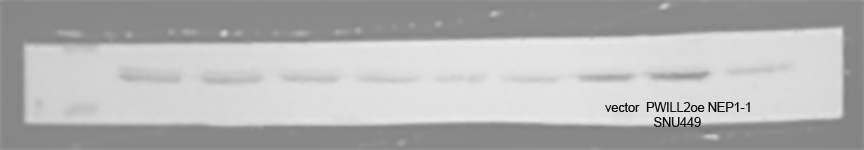


pSTAT3 SNU449 and SNU398


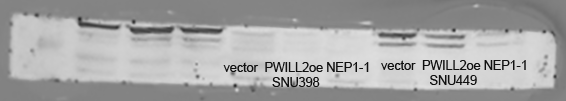


GAPDH


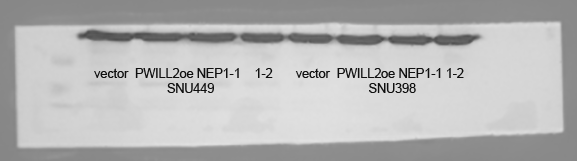

Supplement: Supplementary file 2 — Supplementary Material 2. [file 44342_2025_60_MOESM2_ESM.docx]
